# Air pollution and biomarkers of cardiovascular disease and inflammation in the Malmö Diet and Cancer cohort

**DOI:** 10.1186/s12940-022-00851-1

**Published:** 2022-04-12

**Authors:** Mehjar Azzouz, Yiyi Xu, Lars Barregard, Björn Fagerberg, Bengt Zöller, Peter Molnár, Anna Oudin, Mårten Spanne, Gunnar Engström, Leo Stockfelt

**Affiliations:** 1 Occupational and Environmental Medicine, School of Public Health and Community Medicine, Institute of Medicine, Sahlgrenska Academy, University of Gothenburg, Gothenburg, Sweden; 2 Department of Molecular and Clinical Medicine, Institute of Medicine, Sahlgrenska Academy, University of Gothenburg, Gothenburg, Sweden; 3 Center for Primary Health Care Research, Lund University/Region Skåne, Malmö, Sweden; 4 Department of Occupational and Environmental Medicine, Sahlgrenska University Hospital, Gothenburg, Sweden; 5 Occupational and Environmental Medicine, Department for Laboratory Medicine, Lund University, Lund, Sweden; 6 Division of Sustainable Health, Umeå University, Umeå, Sweden; 7 Environment Department, City of Malmö, Malmö, Sweden; 8 Department of Clinical Sciences in Malmö, CRC, Lund University, Lund, Sweden

**Keywords:** Air pollution, Biomarkers, Cardiovascular disease, Inflammation, C-reactive protein, Particulate matter

## Abstract

**Introduction:**

Air pollution is associated with increased risk of cardiovascular disease, possibly through chronic systemic inflammation that promotes the progression of atherosclerosis and the risk of cardiovascular events. This study aimed to investigate the associations between air pollution and established biomarkers of inflammation and cardiovascular disease.

**Methods:**

The Cardiovascular Subcohort of the Malmö Diet and Cancer cohort includes 6103 participants from the general population of Malmö, Sweden. The participants were recruited 1991–1994. Annual mean residential exposure to particulate matter < 2.5 and < 10 μm (PM_2.5_ and PM_10_), and nitrogen oxides (NO_x_) at year of recruitment were assigned from dispersion models. Blood samples collected at recruitment, including blood cell counts, and biomarkers (lymphocyte- and neutrophil counts, C-reactive protein (CRP), soluble urokinase-type plasminogen activator receptor (suPAR), lipoprotein-associated phospholipase A_2_ (Lp-PLA_2_), ceruloplasmin, orosomucoid, haptoglobin, complement-C3, and alpha-1-antitrypsin) were analyzed. Multiple linear regression models were used to investigate the cross-sectional associations between air pollutants and biomarkers.

**Results:**

The mean annual exposure levels in the cohort were only slightly or moderately above the new WHO guidelines of 5 μg/m^3^ PM_2.5_ (10.5 μg/m^3^ PM_2.5_). Residential PM_2.5_ exposure was associated with increased levels of ceruloplasmin, orosomucoid, C3, alpha-1-antitrypsin, haptoglobin, Lp-PLA_2_ and the neutrophil-lymphocyte ratio. Ceruloplasmin, orosomucoid, C3 and alpha-1-antitrypsin were also positively associated with PM_10_. There were no associations between air pollutants and suPAR, leukocyte counts or CRP. The associations between particles and biomarkers were still significant after removing outliers and adjustment for CRP levels. The associations were more prominent in smokers.

**Conclusion:**

Long-term residential exposure to moderate levels of particulate air pollution was associated with several biomarkers of inflammation and cardiovascular disease. This supports inflammation as a mechanism behind the association between air pollution and cardiovascular disease.

**Supplementary Information:**

The online version contains supplementary material available at 10.1186/s12940-022-00851-1.

## Introduction

Air pollution has been linked to numerous adverse cardiovascular disease events [1]. Air pollution has been demonstrated to increase the risk of myocardial infarction, cerebrovascular disease, heart failure, and hypertension [1, 2]. However, the underlying biomolecular mechanisms contributing to these adverse effects are not fully understood. The evolving body of evidence suggests that oxidative stress and inflammation is a primary underlying mechanism [3, 4]. It is hypothesized that the pulmonary inflammation caused by air pollution subsequently leads to systemic inflammation which over the long-term increases the risk of cardiovascular disease. Another hypothesis is focused on effects of air pollution on the autonomic nervous system, leading to dysregulation of the cardiovascular system. Furthermore, penetration of particulate matters (PM) into the systemic circulation might also directly cause systemic inflammation [1, 2, 5].

C-reactive protein (CRP), soluble urokinase-type plasminogen activator receptor (suPAR), neutrophil-lymphocyte ratio (NLR), lipoprotein-associated phospholipase A_2_ (Lp-PLA_2_), ceruloplasmin, haptoglobin, orosomucoid, complement component 3 (C3) and alpha-1-antitrypsin are all inflammatory biomarkers reported to be associated with cardiovascular disease. Both CRP and ceruloplasmin are established as markers of cardiovascular disease severity and future cardiovascular events, where ceruloplasmin has been shown to be a better predictor of long-term cardiovascular disease risk compared to CRP [6–8]. SuPAR, and Lp-PLA_2_ have also been used as prognostic markers for cardiovascular disease and low-grade inflammation [9, 10]. NLR has been suggested to be a more sensitive marker of inflammation than a non-differentiated cell count or isolated lymphocyte- and neutrophil counts [11]. CRP, ceruloplasmin, haptoglobin, orosomucoid, C3 and alpha-1-antitrypsin are also acute phase proteins with different functions within the immune system [12, 13]. For example, alpha-1-antitrypsin has mostly been studied because of its deficiency being associated with lung tissue degradation [14]. Lp-PLA_2_ travels along low-density lipoprotein and is also expressed by macrophages and other leukocytes in atherosclerotic plaques [10]. For suPAR, a cell surface receptor, its blood level has been shown to reflect immune activation and it has been proposed as a prognostic marker in cardiovascular disease, cancer, and infections [9]. Haptoglobin, alpha-1-antitrypsin and orosomucoid are reflected in the glycA signal, which has been shown to be an independent and more sensitive marker of inflammation and cardiovascular disease compared to CRP [15].

Most epidemiological studies exploring the role of air pollution and inflammation have used CRP or fibrinogen as their main markers of inflammation [3, 16, 17], but some have also used inflammatory cells and cytokines as markers [18–21]. Even though a pooled analysis of studies investigating air pollution and CRP found a positive association, there is large variation in strength and heterogeneity between studies [20, 22]. There are few previous studies focused on other biomarkers of inflammation and cardiovascular disease [23, 24]. The aim of this study was to investigate the association between long-term air pollution exposure and the above-mentioned biomarkers.

## Materials and method

### Study population

The Malmö Diet and Cancer (MDC) cohort is a population-based cohort originally created to study if dietary habits were associated with cancer. All residents in Malmö, Sweden born between 1923 and 1950 without exclusions were invited to an initial screening 1991–1996 and 30,446 participated [25]. A random selection from the MDC were invited 1991–1994 for further clinical testing and collection of blood samples, the so-called cardiovascular subcohort (MDC-CC). The study population of the current study consists of the 6103 participants from the MDC-CC who left fasting blood samples for analysis. Questionnaires about education, occupation, smoking, alcohol intake, dietary habits and other life-style factors were collected at recruitment, and a physical examination was performed, and blood samples were taken. All participants provided written consent. The study was approved by the Regional Ethics Committee at the University of Lund (dnr 2016/4).

### Biomarkers of cardiovascular disease and inflammation

Fasting blood samples were drawn at recruitment. Laboratory markers were analysed at the Department of Clinical Chemistry, Skåne University Hospital, Malmö (SWEDAC approved according to European norm 45,001). Leukocytes, expressed as a total leukocyte count and differentiated into neutrophil- and lymphocyte count were measured from fresh whole blood using the SYSMEX K1000 automatic counter (Sysmex Europe, Norderstedt, Germany). Blood samples were also separated into plasma and serum, and frozen at − 80 °C until analysis of biomarkers of inflammation and cardiovascular disease [26, 27]. CRP levels were measured using the Tina-quant® CRP latex assay (Roche Diagnostics, Basel, Switzerland) on an ADVIA® 1650 Chemistry System (Bayer Healthcare, New York, NY, USA). SuPAR levels were measured using the ELISA suPARnostic® kit (Virogates, Copenhagen, Denmark) [28]. The second generation PLAQ™ test (diaDEXUS Inc., South San Francisco, CA, US) was used to measure Lp-PLA_2_ concentration [29]. Orosomucoid, haptoglobin, complement-C3, α-1-antitrypsin and ceruloplasmin levels were measured using the Cobas c-systems (Roche Diagnostics GmbH, Germany) [26, 30]. The inter-assay coefficient of variation (CV) and intra-assay CV were below 5% for all biomarkers. The number of participants with missing biomarker concentration data varied between 18 and 1412.

### Air pollution modelling

**Fig. 1:**
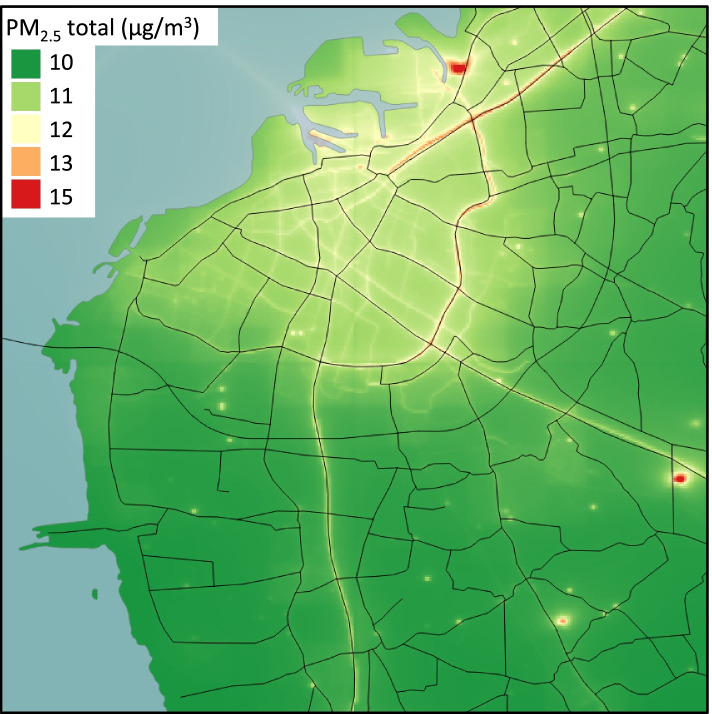
The distribution of PM_2.5_ in the Malmö area in 1992, in μg/m^3^

A Gaussian dispersion model (AERMOD), in conjunction with an emission database (EDB) for Malmö and the surrounding county, was used to determine levels of ambient air pollution [31]. The area covered was 18 km × 18 km with a resolution of 50 m × 50 m (Fig. 1). Annual averages were modelled for the years 1992 and 2000. In between these years air pollution levels were interpolated linearly, and also adjusted for year-to-year variations in meteorology using a ventilation factor. Air pollution levels between 1990 and 1991 were extrapolated using the linear trend of the modelled years. Point and area sources were taken from the EDB. Air pollution levels from road traffic were calculated using the HBEFA (Handbook Emission Factor Road Transport) version 3.3 [31]. Different vehicle types, speeds and driving conditions were factors included in the model.

The different air pollutants modelled were PM_2.5_, PM_10_ and NO_x_. PM_2.5_ and PM_10_ included particulate matter < 2.5 μm and < 10 μm respectively. PM coarse included particulate matter between 2.5 μm and 10 μm. Local emissions of each air pollutant were the sum of road traffic exhaust, road traffic wear, residential heating, shipping, and other household and industry emissions. The sum of road traffic exhaust and road traffic wear formed the traffic-related PM_10_, so called “PM_10_ traffic,” and “PM_10_ non-traffic” included local PM_10_ emissions from all other sources. Then, the total exposure to each air pollutant was calculated as the local emission plus the long-range transport (LRT). LRT was estimated by taking the difference between the measured background concentration in the central urban monitoring station in Malmö (“Rådhuset”) and the modelled contribution from all local sources at this monitoring station. LRT is primarily from countries and areas south of Sweden, such as the metropolitan area of Copenhagen, Denmark, and central Europe. This paper focuses on total exposure to PM_2.5_, PM_10_, PM coarse and NO_x_ as well as local source-specific PM_10_ traffic and PM_10_ non-traffic.

The annual addresses for individuals were collected from Statistics Sweden for the years 1990 to 1994 and were geocoded automatically based on the location of the building entrance. Then, each participant was assigned exposure levels for the different air pollutants for the year of recruitment, using QGIS version 3.10.3 (QGIS development team, 2020). There were 46 participants for which air pollution exposure at recruitment could not be assigned, and they were therefore excluded from further analysis.

### Covariates

All covariates were collected at recruitment. Systolic blood pressure (mmHg) was measured by using a mercury-column sphygmomanometer. Use of non-steroidal anti-inflammatory drugs (NSAID) and statins was obtained through the questionnaire and dichotomized according to their respective anatomical therapeutic chemical codes (ATC-codes). Prevalent diabetes and prevalent cardiovascular disease (CVD) were retrieved from the National Patient Register, together with self-reported disease or use of medication from the questionnaire. Prevalent CVD was defined as history of coronary event, heart failure event or stroke. Body mass index (BMI) was calculated by dividing measured weight (kg) by squared height (m). Apo-A_1_ and Apo-B were obtained from the collected fasting blood samples. Education was divided into three categories: low (1–9 years of education), intermediate (10–12 years) and high (> 12 years). Occupations from the questionnaire were categorized into three groups according to the Swedish socioeconomic classification: blue-collar, white-collar, and self-employed/farmers/others. Self-reported smoking status used four categories: regular smoker, occasional smoker, previous smoker, and never-smoker. For current smokers (i.e., regular and occasional smokers), information on number of cigarettes per day was also collected (for non-smokers set as 0). Country of birth was categorized into those born in Sweden versus outside of Sweden. Self-reported alcohol consumption was calculated as grams/day. Leisure-time physical activity was defined using an aggregate score based on activity based on both intensity and time. Information about living with a partner (irrespective of marital status) was obtained through the questionnaire. The mean area income, as an index of area level socioeconomic status, was collected for the year 1994 from Statistics Sweden, where the areas are divided into Small Areas for Marketing Statistics (SAMS).

### Statistical analysis

Descriptive statistics for covariates, biomarker concentrations and air pollution exposures for all participants were calculated as counts and the proportion of sample for categorical variables, means (standard deviation) for normally distributed continuous variables and median (5–95%) for non-normally distributed continuous variables. The correlation between the biomarkers and between the air pollution exposures was evaluated using Spearman’s correlation coefficient.

The biomarker concentrations had log-linear distributions when examining histograms of relative frequency for the biomarker concentrations and were log-transformed by the natural logarithm. Multiple linear regression was used to examine the association between the air pollution exposures and ln-transformed biomarker concentrations. The main analysis used air pollutants as continuous variables. To test for a possible non-linear association, supplementary analysis was also performed by categorizing air pollutants into quartiles. Three covariate models were used for each association studied, based on a directed acyclic graph (DAG) using a priori assumptions of causal relationships. The crude model (M0) was adjusted for age and sex. The main model (M1) further adjusted for alcohol consumption, BMI, smoking status, and education as these were determined to be likely confounders and suggested as the minimal adjustment set for the total effect according to the DAG (Additional file 1). The full model (M2) additionally included number of cigarettes, mean area income, occupational class, leisure-time physical activity, systolic blood pressure, Apo-B/Apo-A1 ratio, prevalent diabetes, country of birth, cohabitation status, statin use and NSAID use. These covariates were considered risk factors for inflammation or cardiovascular disease, potential mediators (blood pressure and diabetes) or confounders that were not suggested in the minimal adjustment set by the DAG. For each analysis, there was a loss of subjects due to missing covariates of approximately 3.5% between M0 and M1 and an additional 5% between M1 and M2.

A series of sensitivity analyses were performed using the main model with total PM_2.5_ as the exposure: 1) a regression model with CRP as a further covariate together with each of the other biomarkers; 2) an analysis that excluded individuals with extreme biomarker levels (i.e., above the 95th percentile); 3) models with further adjustment for the sampling season, divided into winter (December–February, spring (March–May), summer (June–August), and autumn (October–December); 4) a model using Poisson regression with the number of biomarkers above the 75% percentile for each participant as the outcome; 5) an analysis that excluded individuals with diabetes; 6) an analysis that excluded individuals with CVD; 6) a model where biomarkers are grouped using principle component analysis (PCA).

For the main covariate model and PM_2.5_ exposure, a stratified analysis was performed for age (below or above 57, the median age of the study population), sex and smoking status (never or ever smokers). Furthermore, potential effect modification of the association between PM_2.5_ and biomarkers by the covariates included in the M1 was evaluated by introducing interaction terms into the model. All analyses used an alpha level of 0.05 and were done using STATA 16.1 (StataCorp, Texas, TX, USA).

## Results

Characteristics of the study population, together with air pollution levels are presented in Table 1. The air pollution levels in each quartile are presented in the Additional file 2. The mean age of the study population was 58 years at recruitment. Slightly more women than men participated, and most were born in Sweden. About one quarter were current smokers (i.e., regular or occasional smokers) and few regularly used statins and/or NSAIDs. Levels of air pollution exposure were moderate; the mean total PM_2.5_ was 10.5 μg/m^3^, out of which 1.5 μg/m^3^ was locally emitted and 9.0 μg/m^3^ was from LRT. This is moderately above the new WHO guidelines of 5 μg/m^3^ of annual average PM_2.5_ [32]. Levels of biomarker concentrations were mostly in the normal range. Biomarker concentrations were not significantly associated with the sampling season (*p* > 0.1 for all biomarkers).

The correlations between air pollutants were high, with Spearman’s correlation coefficients above 0.5 for all (Additional file 3). Correlations between the biomarkers are presented in Additional file 4. The correlations between CRP and the other biomarkers were generally low except for orosomucoid (*rs* = 0.51), C3 (rs = 0.44) and haptoglobin (*rs* = 0.43) (Table 1).

**Table 1:** Characteristics of study population, exposure levels and biomarker levels at recruitment

	All *n* = 6103	Missing (n)
Age, years (mean, SD)	57.5 (5.9)	1
Sex (n (%))		0
Men	2572 (42%)	
Women	3531 (57%)	
Alcohol, g (mean, SD)	10.4 (12.5)	323
Smoking status (n (%))		315
Regular	1347 (23%)	
Occasional	273 (5%)	
Previous smoker	1915 (33%)	
Never smoker	2253 (39%)	
Number of cigarettes per day for regular and occasional smokers only (mean, SD)	14 (9)	185
Physical activity score (mean, SD)	8137 (5923)	366
Living alone vs with partner (n (%))		315
Living alone	1345 (23%)	
With partner	4443 (77%)	
Country of birth (n (%))		314
Sweden	5125 (89%)	
Outside of Sweden	664 (12%)	
Area Income 1994, Euro (mean, SD)	13,830 (3047)	33
Education (n (%))		322
Low (< 9)	2676 (47%)	
Moderate	1982 (34%)	
High (> 12)	1123 (19%)	
Occupation (n (%))		355
Blue collar	2241 (39%)	
White collar	2906 (51%)	
Others	601 (11%)	
Systolic blood pressure mmHg (mean, SD)	141 (19)	1
Diabetes (n (%))		0
Yes	293 (5%)	
No	5810 (95%)	
CVD (n (%))		0
Yes	151 (2%)	
No	5951 (98%)	
BMI^a^, kg/m2 (mean, SD)	25.8 (4)	8
Apo-B/Apo-A_1_ (mean, SD)	0.7 (0.2)	204
Statin use (n (%))		0
Yes	28 (0.5%)	
No	6075 (99.5%)	
NSAID use (n (%))		0
Yes	63 (1%)	
No	6040 (99%)	
Total PM_10,_ μg/m^3^ (mean, SD)	14.9 (1.7)	46
Total PM_2.5,_ μg/m^3^ (mean, SD)	10.5 (0.8)	46
Total PM coarse, μg/m^3^ (mean, SD)	4.4 (1.1)	46
Total NO_x_ μg/m^3^ (mean, SD)	39.1 (13.8)	46
PM_10_ traffic, μg/m^3^ (mean, SD)	2.8 (1.3)	46
PM_10_ non-traffic, μg/m^3^ (mean SD)	0.9 (0.2)	46
Leukocytes, # cells × 10^9^/L (median, p_5_ – p_95_)	5.9 (3.9–9)	18
NLR (median, p_5_ – p_95_)^b^	2 (1.1–3.7)	36
CRP, mg/L (median, p_5_ – p_95_)^c^	1.4 (0.3–8.4)	799
SuPAR, ng/ml (median, p_5_ – p_95_)	2.8 (1.9–4.8)	838
Lp-PLA_2_, ng/ml (median, p_5_ – p_95_)	256 (157–415)	710
Ceruloplasmin, g/L (median, p_5_ – p_95_)	0.49 (0.35–0.73)	1248
Orosomucoid, g/L (median, p_5_ – p_95_)	0.7 (0.4–1.1)	970
Haptoglobin, g/L (median, p_5_ – p_95_)	1.3 (0.5–2.3)	1412
C3, g/L (median, p_5_ – p_95_)	1.51 (1.1–2.2)	909
Alpha-1-antitrypsin, g/L (median, p_5_ – p_95_)	1.2 (0.8–1.7)	1005

Means are presented with SD, medians with a 5–95% range and counts with percentages of subjects

^a^Body Mass Index

^b^Neutrophil-lymphocyte ratio

^c^Median of CRP was 1.4 mg/L

The associations between exposure to air pollutants and biomarkers are presented in Tables 2 and 3. Increased levels of ceruloplasmin, orosomucoid, C3 and alpha-1-antitrypsin were consistently associated with higher levels of most air pollutants (PM_2.5_, PM_10_, PM coarse and PM_10_ non-traffic). For PM_2.5_ and PM_10_, the associations were robust after adjusting for different covariates, while for PM coarse and PM_10_ non-traffic the associations were only borderline significant in M1 and no longer significant in M2.

**Table 2:** Association between total PM_2.5_, PM_10_ as well as NO_x_ exposure and biomarkers

		PM_2.5_		PM_10_		NO_x_	
Increment of exposure		5 μg/m^3^		5 μg/m^3^		20 μg/m^3^	
		β-Coefficient (95% CI)	*P* value*	β-Coefficient (95% CI)	P value*	β-Coefficient (95% CI)	P value*
Leukocytes	M0	−0.021 (− 0.064–0.022)	0.34	0.017 (− 0.0023–0.036)	0.084	**0.015 (0.0057–0.024)**	**0.002**
	M1	−0.02 (− 0.062–0.021)	0.33	0.0004 (− 0.018–0.019)	0.97	0.0024 (− 0.0067–0.011)	0.61
	M2	− 0.095 (− 0.38–0.19)	0.52	−0.0062 (− 0.026–0.014)	0.55	−0.0022 (− 0.012–0.0078)	0.67
NLR	M0	**0.076 (0.013–0.14)**	**0.019**	**0.03 (0.0018–0.059)**	**0.037**	0.0098 (− 0.004–0.024)	0.17
	M1	**0.066 (0–0.131)**	**0.05**	0.023 (− 0.0062–0.053)	0.12	0.0062 (− 0.0081–0.021)	0.4
	M2	**0.074 (0.0054–0.14)**	**0.034**	0.03 (− 0.0031–0.062)	0.076	0.0074 (− 0.0089–0.024)	0.37
CRP	M0	−0.0438 (− 0.2361–0.1485)	0.655	0.047 (− 0.04–0.13)	0.29	0.035 (− 0.0065–0.077)	0.097
	M1	0.0621 (− 0.1216–0.2459)	0.507	0.038 (− 0.045–0.12)	0.37	0.015 (− 0.025–0.055)	0.46
	M2	0.0393 (− 0.1502–0.2288)	0.684	0.0034 (− 0.087–0.094)	0.94	−0.0075 (− 0.053–0.038)	0.74
SuPAR	M0	− 0.023 (− 0.0755–0.0294)’	0.389	0.022 (− 0.0012–0.045)	0.063	**0.018 (0.0069–0.029)**	**0.002**
	M1	−0.0173 (− 0.067–0.0323)	0.494	0.0053 (− 0.017–0.027)	0.64	0.0062 (− 0.0045–0.017)	0.26
	M2	−0.0199 (− 0.0704–0.0306)	0.441	−0.0024 (− 0.026–0.022)	0.84	0.0019 (− 0.01–0.014)	0.76
LP-PLA2	M0	**0.0896 (0.0365–0.1427)**	**0.001**	0.019 (− 0.0046–0.043)	0.11	0.00086 (− 0.011–0.012)	0.88
	M1	**0.0846 (0.0311–0.1382)**	**0.002**	0.011 (− 0.014–0.035)	0.39	−0.0045 (− 0.016–0.0071)	0.45
	M2	**0.085 (0.0303–0.1397)**	**0.002**	0.017 (− 0.009–0.043)	0.2	−0.00027 (− 0.013–0.013)	0.97
Ceruloplasmin	M0	**0.1319 (0.0909–0.1729)**	**< 0.001**	**0.043 (0.025–0.061)**	**< 0.001**	0.0082 (− 0.00056–0.017)	0.067
	M1	**0.1369 (0.0954–0.1784)**	**< 0.001**	**0.043 (0.024–0.061)**	**< 0.001**	0.0072 (− 0.0017–0.016)	0.11
	M2	**0.1328 (0.0898–0.1757)**	**< 0.001**	**0.039 (0.019–0.059)**	**< 0.001**	0.0025 (− 0.0075–0.012)	0.62
Orosomucoid	M0	**0.186 (0.1312–0.2408)**	**< 0.001**	**0.056 (0.032–0.08)**	**< 0.001**	0.0082 (− 0.0035–0.02)	0.17
	M1	**0.2045 (0.1508–0.2582)**	**< 0.001**	**0.053 (0.029–0.077)**	**< 0.001**	0.004 (− 0.0074–0.015)	0.49
	M2	**0.205 (0.1505–0.2595)**	**< 0.001**	**0.052 (0.026–0.078)**	**< 0.001**	− 0.00097 (− 0.014–0.012)	0.88
Haptoglobin	M0	**0.1046 (0.0154–0.1937)**	**0.022**	**0.053 (0.014–0.092)**	**0.008**	**0.024 (0.0052–0.043)**	**0.013**
	M1	**0.1098 (0.0222–0.1974)**	**0.014**	0.038 (− 0.0004–0.077)	0.058	0.013 (− 0.0059–0.032)	0.18
	M2	**0.0981 (0.0075–0.1886)**	**0.034**	0.026 (− 0.016–0.069)	0.23	0.0043 (− 0.017–0.025)	0.69
C3	M0	**0.1427 (0.1017–0.1837)**	**< 0.001**	**0.043 (0.025–0.061)**	**< 0.001**	0.0064 (− 0.0023–0.015)	0.15
	M1	**0.1686 (0.1302–0.2071)**	**< 0.001**	**0.047 (0.03–0.064)**	**< 0.001**	0.0066 (− 0.0015–0.015)	0.11
	M2	**0.1618 (0.1228–0.2007)**	**< 0.001**	**0.042 (0.024–0.06)**	**< 0.001**	0.00048 (− 0.0085–0.0095)	0.92
alpha-1-antitrypsin	M0	**0.1196 (0.0734–0.1658)**	**< 0.001**	**0.043 (0.023–0.064)**	**< 0.001**	**0.01 (0.00033–0.02)**	**0.043**
	M1	**0.1143 (0.0677–0.161)**	**< 0.001**	**0.037 (0.017–0.058)**	**< 0.001**	0.007 (− 0.0029–0.017)	0.17
	M2	**0.105 (0.0564–0.1537)**	**< 0.001**	**0.029 (0.006–0.052)**	**0.013**	0.00042 (− 0.011–0.012)	0.94

*p < 0.05 bolded. M0: adjusted for age and sex. M1: adjusted for age, sex, alcohol consumption, BMI, smoking status, and education. M2: adjusted for age, sex, alcohol consumption, BMI, smoking status, education, number of cigarettes, mean area income, occupational class, leisure-time physical activity, systolic blood pressure, Apo-B/Apo-A_1_ ratio, prevalent diabetes, country of birth, cohabitation, statin use and NSAID use

**Table 3:** Association between PM coarse, PM_10_ traffic and PM_10_ non-traffic exposure and biomarkers

		PM coarse		PM_10_ traffic		PM_10_ non-traffic	
Increment of exposure		5 μg/m^3^		1 μg/m^3^		1 μg/m^3^	
		β-Coefficient (95% CI)	P value*	β-Coefficient (95% CI)	P value*	β-Coefficient (95% CI)	P value*
Leukocytes	M0	0.01 (− 0.016–0.036)	0.27	**0.0081 (0.0033–0.013)**	**0.001**	0.0067 (− 0.033–0.046)	0.74
	M1	0.01 (− 0.018–0.038)	0.47	0.0012 (− 0.0034–0.0059)	0.6	−0.0064 (− 0.044–0.031)	0.74
	M2	− 0.0031 (− 0.034–0.028)	0.85	− 0.0013 (− 0.0065–0.004)	0.64	−0.013 (− 0.052–0.025)	0.5
NLR	M0	0.034 (− 0.0089–0.077)	0.12	0.005 (− 0.0022–0.012)	0.17	0.027 (− 0.031–0.085)	0.36
	M1	0.023 (− 0.022–0.067)	0.32	0.0031 (− 0.0042–0.011)	0.41	0.011 (− 0.049–0.07)	0.73
	M2	0.031 (− 0.02–0.081)	0.24	0.0039 (− 0.0046–0.012)	0.37	0.0057 (− 0.057–0.069)	0.86
CRP	M0	0.13 (− 0.004–0.26)	0.058	0.019 (− 0.0024–0.041)	0.082	−0.086 (− 0.26–0.087)	0.33
	M1	0.057 (− 0.067–0.182)	0.37	0.0057 (− 0.015–0.026)	0.59	0.0017 (− 0.16–0.17)	0.98
	M2	−0.014 (− 0.15–0.13)	0.85	−0.0074 (− 0.031–0.016)	0.54	−0.046 (− 0.22–0.13)	0.61
SuPAR	M0	**0.059 (0.025–0.094)**	**0.001**	**0.011 (0.0052–0.017)**	**< 0.001**	−0.017 (− 0.064–0.03)	0.48
	M1	0.019 (− 0.014–0.053)	0.25	0.0039 (− 0.0016–0.0093)	0.17	−0.0089 (− 0.053–0.035)	0.69
	M2	0.0048 (− 0.032–0.042)	0.8	0.0016 (− 0.0045–0.0077)	0.61	−0.011 (− 0.057–0.034)	0.63
LP-PLA_2_	M0	0.0028 (− 0.033–0.038)	0.88	0.0008 (− 0.0052–0.0067)	0.8	0.0056 (− 0.042–0.053)	0.82
	M1	−0.015 (− 0.051–0.021)	0.42	−0.0023 (− 0.0083–0.0036)	0.44	0.0051 (− 0.043–0.053)	0.84
	M2	− 0.0053 (− 0.046–0.035)	0.8	−0.0003 (− 0.007–0.0065)	0.94	0.026 (− 0.024–0.076)	0.31
Ceruloplasmin	M0	**0.039 (0.012–0.066)**	**0.005**	0.0042 (− 0.0003–0.0087)	0.065	0.035 (− 0.0014–0.072)	0.06
	M1	**0.036 (0.0083–0.063)**	**0.011**	0.0035 (− 0.001–0.0081)	0.13	**0.039 (0.0021–0.076)**	**0.038**
	M2	0.024 (− 0.0071–0.055)	0.13	0.001 (− 0.0042–0.0061)	0.71	0.026 (− 0.013–0.065)	0.18
Orosomucoid	M0	**0.045 (0.0088–0.081)**	**0.015**	0.012 (− 0.0031–0.027)	0.12	0.032 (− 0.016–0.081)	0.19
	M1	0.03 (− 0.0054–0.065)	0.097	0.002 (− 0.0038–0.0079)	0.5	**0.05 (0.0027–0.097)**	**0.038**
	M2	0.017 (− 0.022–0.056)	0.39	−0.0013 (− 0.017–0.015)	0.87	0.046 (− 0.0032–0.095)	0.067
Haptoglobin	M0	**0.073 (0.014–0.13)**	**0.015**	**0.012 (0.0025–0.022)**	**0.014**	0.026 (− 0.053–0.11)	0.52
	M1	0.038 (− 0.02–0.096)	0.2	0.006 (− 0.0036–0.016)	0.22	0.029 (− 0.049–0.11)	0.46
	M2	0.011 (− 0.055–0.076)	0.75	0.0014 (− 0.0095–0.012)	0.8	0.013 (− 0.069–0.094)	0.76
C3	M0	**0.034 (0.0073–0.061)**	**0.013**	0.0037 (− 0.0007–0.0081)	0.1	0.0047 (− 0.032–0.041)	0.8
	M1	**0.033 (0.0076–0.058)**	**0.011**	0.0032 (− 0.001–0.0074)	0.14	0.033 (− 0.0008–0.067)	0.055
	M2	0.016 (− 0.012–0.044)	0.26	−0.0001 (− 0.0048–0.0045)	0.96	0.022 (− 0.013–0.057)	0.21
alpha-1-antitrypsin	M0	**0.045 (0.014–0.075)**	**0.004**	**0.0055 (0.0004–0.011)**	**0.033**	0.035 (− 0.0059–0.076)	0.094
	M1	**0.033 (0.0026–0.064)**	**0.034**	0.0036 (− 0.0015–0.0087)	0.17	0.032 (−0.0086–0.074)	0.12
	M2	0.014 (−0.021–0.049)	0.44	−0.0001 (− 0.006–0.0057)	0.97	0.019 (− 0.025–0.062)	0.39

*p < 0.05 bolded. M0: adjusted for age and sex. M1: adjusted for age, sex, alcohol consumption, BMI, smoking status, and education. M2: adjusted for age, sex, alcohol consumption, BMI, smoking status, education, number of cigarettes, mean area income, occupational class, leisure-time physical activity, systolic blood pressure, ApoA/ApoB1 ratio, prevalent diabetes, country of birth, cohabitation, statin use and NSAID use

Haptoglobin, Lp-PLA_2_ and NLR were positively associated with PM_2.5_ in all covariate models. Haptoglobin was also associated with PM_10_, NO_x_, PM coarse, and PM_10_ traffic, and NLR was associated with PM_10_ in M0.

No association with air pollutants was found for the two most widely clinically used inflammatory biomarkers, i.e., leukocyte counts and CRP, or suPAR in M1, apart from some positive associations in the minimally adjusted M0 model.

The magnitudes of increase in biomarker concentrations were moderate but not negligible. For instance, per 5 μg/m^3^ increase in total PM_2.5_ exposure there were: 15% increase in ceruloplasmin, 23% in orosomucoid, 18% in C3, 12% in alpha-1-antitrypsin, 12% in haptoglobin, 9% in Lp-PLA_2_, and 7% increase in NLR.

For air pollutants categorized in quartiles, the results were generally consistent with those from the linear models (Additional file 7). For ceruloplasmin, orosomucoid and C3, significantly increased levels were found in quartiles 2–4 of PM_2.5_ and PM_10_ exposure, compared to the first quartile, with a linear trend. For alpha-1-antitrypsin, the associations with PM_2.5_ and PM_10_ were only significant in the third and fourth quartiles. An increased level of suPAR was found in the third quartile of NO_x_, PM_10_ traffic compared to the first quartile, but not in the highest quartile, possibly indicating a non-monotonic relationship. This pattern was not seen for other air pollutants, however.

The results from the sensitivity analyses using PM_2.5_ exposure and the M1 covariate model are presented in Additional file 5. After adjustment for CRP levels and after excluding those with CVD, the results for most of the biomarkers remained the same except for NLR, where the association became non-significant. After excluding individuals with biomarker concentrations above the 95th percentile, the results were similar to the main analysis but with slightly lower beta estimates. Adjusting for seasons and excluding those with diabetes did not change the results. An analysis with number of biomarkers above the 75% percentile of each biomarker gave a significant incidence rate ratio (IRR 1.03, *p* = 0.001). After grouping biomarkers into two components using PCA and running the model, the results indicated that the first component (including CRP, ceruloplasmin, orosomucoid, haptoglobin, C3 and alfa-1-antitrypsin) was associated with air pollution exposure while the second component (including leukocytes, NLR, suPAR and LP-PLA_2_) was not.

The results from stratified analyses and analysis of interactions using PM_2.5_ exposure and the M1 covariate model are presented in Additional file 6. We found indications of effect modification by sex, with stronger associations for PM_2.5_ and ceruloplasmin and orosomucoid in women. Stratified by sex, the associations between PM_2.5_ and ceruloplasmin, orosomucoid, C3 and alpha-1-antitrypsin were significant in both men and women however, the associations with NLR and Lp-PLA_2_ only in men, and the association for haptoglobin only in women. There was no significant effect modification by age, but in the stratified analyses the association for Lp-PLA_2_ was significant only in older participants, and for haptoglobin only in the younger participants. For smoking status the associations between PM_2.5_ and alpha-1-antitrypsin and ceruloplasmin were stronger in never-smokers than in smokers. The associations with PM_2.5_ were significant for both smokers and never-smokers for both ceruloplasmin, orosomucoid, C3, alpha-1-antitrypsin and Lp-PLA_2_, however, and for NLR and haptoglobin only significant in smokers.

## Discussion

In this large population-based Swedish cross-sectional study with average exposure levels slightly above the WHO limit values, we found clear associations between exposure to one-year average residential ambient air pollution and biomarkers of inflammation and cardiovascular disease. The associations were most consistent for PM_2.5_ exposure, which was associated with increased levels of ceruloplasmin, orosomucoid, C3, alpha-1-antitrypsin, haptoglobin, Lp-PLA_2_ and the neutrophil-lymphocyte ratio. The associations were only marginally affected by adjustment for potential confounders and mediators, and persisted in sensitivity analyses. For the clinically more commonly used biomarkers CRP and leukocyte count, however, there were no significant associations with any air pollutant.

We observed positive associations between PM exposure and orosomucoid, haptoglobin and C3**.** For these biomarkers, few previous studies have investigated the association with long-term exposure to air pollution. In an ecological study, Stiller-Winkler et al. (1996) investigated the difference in 16 serum protein concentrations between the inhabitants in the more polluted area of Cologne and the control area Borken in Germany in a sample of 500 women and found significantly higher levels of C3, haptoglobin and orosomucoid, but not of ceruloplasmin, alpha-1 antitrypsin and CRP in the polluted area. However, only SO_2_ and NO_2_ differed between the two areas while PM did not [33], and in the current study we found associations with PM but not with NO_x_. In another study performed in 480 school-aged children exposed to environmental tobacco smoke, a source of PM exposure [34], Shima and Adachi (1996) reported increased levels of C3 and haptoglobin, but not ceruloplasmin, associated with exposure [35]. However, the differences in design, age range, exposure sources and levels make it difficult to directly compare our findings with these studies.

While few studies have studied associations between exposure to air pollutants and these biomarkers, a number of studies have been performed to understand their biological function and relation to diseases. The glycA nuclear magnetic resonance (NMR) signal captures the concentrations of circulating glycosylated proteins in the liver among which the late acute phase reactants haptoglobin, alpha-1-antitrypsin and orosomucoid make significant contributions to the GlycA signal [36, 37]. GlycA measured by NMR spectroscopy is associated with disease activity and cardiovascular disease risk in chronic inflammatory diseases [37]. The glycA NMR signal has been shown to be a predictor of cardiovascular disease and diabetes mellitus, independently of the increase in CRP, associated with these conditions. Furthermore, the glycA NMR signal has been linked to various autoimmune diseases such as psoriasis and rheumatoid arthritis [15, 36] for which there are some studies reporting associations with air pollution [38, 39]. These conditions could be areas of interest in future air pollution research. The findings from our study feature a selection of biomarkers other than the widely studied CRP and cell counts to use for investigating health effects of air pollution, potentially providing new insights into the underlying mechanisms of health effects related to air pollution.

Several epidemiological studies, including a meta-analysis, have reported positive associations between air pollution exposure and CRP. However, there is heterogeneity within the studies in regards to study design, study quality, CRP levels and exposure levels [22, 40–42]. Similar positive associations were observed with leukocyte count [40, 43, 44]. In our study, no association was observed for either CRP or leukocyte count. One factor that could explain this difference is that most previous studies were performed at higher exposure levels than those in the present cohort, probably indicating that higher levels are needed to trigger the alterations in CRP and leukocyte count. Interestingly, we found a positive association between PM_2.5_ and NLR, a marker that has been shown to be more sensitive than leukocyte count in the detection of subclinical inflammation [11]. In our sensitivity analysis the associations between PM and the biomarkers were still significant after including CRP as a covariate. Our findings could indicate that a pathway that does not include CRP is important for the cardiovascular health effects of air pollution, and that biomarkers other than CRP are more important when studying this topic.

Although many of the biomarkers in this study, like CRP, are acute phase reactants induced by IL-6, they differ from CRP in that they rise later in the acute phase reaction [12]. A possible explanation for finding positive associations for most biomarkers but not CRP or leukocyte count is thus that for shorter episodes of high air pollution levels the early reactants have only transient increases, while the late reactants with longer half-lives remain increased. When grouping biomarkers using PCA, the first component included CRP, ceruloplasmin, orosomucoid, haptoglobin, C3 and alfa-1-antitrypsin while the second component included leukocytes, NLR, suPAR and LP-PLA_2_. We found that PM_2.5_ exposure was only significantly associated with the first component, further indicating the positive association for the late reactants and interestingly CRP. Our study was not designed to observe acute variations in air pollution exposure and its influence on short-term changes in biomarker concentrations. We used the air pollution at the recruitment year to reflect relatively long-term exposure, and our findings should therefore mirror sub-chronic effects rather than transitory air pollution-related responses of biomarkers.

The effect sizes were moderate, but not negligible. The increase in biomarker concentrations associated with air pollution observed in this study would not lead to a substantial increase in the risk of cardiovascular disease and other diseases related to inflammation for the individual. However, a modest increase in the population mean would have considerable effects on public health. Very high levels of biomarkers of inflammation are likely to be the results of infections or other causes rather than moderate PM exposure. In a sensitivity analysis we thus removed the extreme levels of the biomarkers (top 5%) since we assumed that the highest values for these biomarkers were not due to ambient air pollution. The results remained essentially unchanged.

Our study suggests that relatively long-term exposure to particulate air pollutants is pro-inflammatory also at moderate levels. Among the air pollutants we investigated, the associations were most clear with exposure to PM_2.5_. This is in line with similar studies as well as experimental studies [45, 46]. As PM_2.5_ consists of proportionally more ultrafine particles (< 0.1 μm) compared to PM_10_, the particles can travel deeper in the airways reaching alveoli and even pass into the blood stream. From there, they induce inflammatory reactions, that contribute to the systemic inflammation associated with air pollution [40, 47]. Similar associations with biomarkers were seen for PM_10_ exposure, although not as clearly. A combined increase in multiple biomarkers would point towards a stronger inflammatory response. Conducting a supplementary analysis between PM_2.5_ and the number of biomarkers above the 75% percentile of each biomarker in M1 gave a significant association (*p* = 0.001), further denoting the role of PM_2.5_ in the inflammatory response.

Although we found consistent associations for total PM, no clear associations were observed between NO_x_ and PM traffic and biomarkers in the confounder-adjusted models. A possible explanation could be the difference in exposure contrast, which was much larger for total exposure levels that include all local sources and LRT. Both NO_x_ and PM traffic are highly associated with local traffic emissions. In our study, 20% of the NO_x_ exposure was due to LRT, while LRT made up 76% of PM_10_ exposure and 86% of PM_2.5_ exposure. LRT, mainly from continental Europe and the nearby (28 km) city of Copenhagen, constituted a large proportion of total PM levels in our study area. It could thus be argued that abatement strategies to prevent health effects of air pollution exposure must also include actions on an international level.

A strength of the present study is that many inflammatory and cardiovascular biomarkers were investigated, some of them not previously studied in relation to air pollution, in a relatively large and population-based cohort, with extensive information of personal characteristics and lifestyle factors, which decreases the risk of residual confounding. The differences in outcome between covariate models were small, indicating that the effect of confounders is limited. However, residual confounding cannot be excluded since many factors such as diet and genetics can influence the effect of air pollution on inflammation [48–50]. Since multiple comparisons were made, the risk of false positive findings must be considered. A strict control for multiple testing such as Bonferroni correction would increase other errors of interpretation however [51], especially as the different exposures were highly correlated, so instead we focus the interpretation on the overall pattern rather than the significance level for each specific biomarker and exposure. We also observed exposure-response relationships between air pollutants and biomarkers, with log-linear trends with PM_2.5_ exposure for many biomarkers and no clear non-monotonic trend in analysis by quartiles.

Another strength is the high resolution (50 m × 50 m) exposure modelling, including several sources and pollutant types. However, the correlation between different air pollutants and sources was high (Additional file 3), which made it difficult to separate effects of specific pollutants and sources. Therefore the analysis was only done with single-pollutant models. Furthermore, similar to other large epidemiological studies using air pollution exposure, the estimation of ambient air pollution exposure was based on residential addresses, and not actual personal exposure.

The air pollution levels were somewhat higher in Sweden in the 1990s, when the biomarker samples for the present study were collected, than they are today. The levels were similar to those found today in many European countries and the US [52] and above new WHO guidelines of 5 μg/m^3^ for PM_2.5_ [32], and lower than in many large cities and low-income countries [52]. A limitation, however, is that the exposure levels and contrast were modest. Associations present only at high exposure levels would not be captured by our study. For example, a similar cross-sectional study in China, where exposure is higher, found a significant association with CRP [53].

## Conclusion

In this cross-sectional population-based study, exposure to PM was significantly and positively associated with biomarkers of inflammation and cardiovascular risks, mainly ceruloplasmin, orosomucoid, C3, and alpha-1-antitrypsin, indicating an increased risk at moderate exposure levels. There was also an association between PM_2.5_ exposure and levels of haptoglobin, Lp-PLA_2_ and NLR, whereas CRP and leukocyte count were not associated with exposure to any air pollutant. This study adds to current knowledge of air pollution and biomarkers of inflammation and cardiovascular disease. The association between the biomarkers used in this study and air pollution provides further support for increased inflammation as the mechanism behind the association between air pollution and risk of cardiovascular disease. These biomarkers are of interest in future studies of air pollution, and may provide new insight into the underlying mechanism of cardiovascular effects related to air pollution.

## Supplementary Information

**Additional file 1.**

**Additional file 2.**

**Additional file 3.**

**Additional file 4.**

**Additional file 5.**

**Additional file 6.**

**Additional file 7.**

## Data Availability

The data that support the findings of this study are available from the Lund University Medical Faculty – Malmo Diet and Cancer Cohort, but restrictions apply to the availability of these data, which were used under license for the current study, and are not publicly available. Data are however available from the authors upon reasonable request and with permission of Lund University Medical Faculty – Malmo Diet and Cancer Cohort. Ethical approval from The Swedish Ethical Review Authority is needed to access the data.

## Abbreviations

Apo-B/Apo-A_1_: Ratio between apolipoprotein B and apolipoprotein A_1_
C3: Complement component 3
CRP: C-reactive protein
DAG: Directed acyclic graph
EDB: Emission database
glycA NMR: glyca nuclear magnetic resonance
HBEFA: Handbook Emission Factor Road Transport
Lp-PLA_2_: Lipoprotein-associated phospholipase A_2_
LRT: Long-range transport
MDC: Malmö Diet and Cancer
MDC-CC: Malmö Diet and Cancer cardiovascular subcohort
NLR: Neutrophil-lymphocyte ratio
NO_x_: Nitrogen oxides
NSAID: Non-steroidal anti-inflammatory drugs
PM: Particulate matters
PM coarse: Particulate matter < 10 μm and > 2.5 μm
PM_10_: Particulate matter with an aerodynamic diameter of < 10 μm
PM_10_ non-traffic: Non-traffic related PM_10_
PM_10_ traffic: Traffic related PM_10_
PM_2.5_: Particulate matter with an aerodynamic diameter of < 2.5 μm
SAMS: Small Areas for Marketing Statistics
suPAR: Soluble urokinase-type plasminogen activator receptor
WHO: World Health Organization
PCA: Principal component analysis
