# Supplementary material for: Air pollution and biomarkers of cardiovascular disease and inflammation in the Malmö Diet and Cancer cohort
Source: Environ Health. 2022 Apr 12;21:39. doi: 10.1186/s12940-022-00851-1 (PMC9004064; doi:10.1186/s12940-022-00851-1)
Supplement: Supplementary file 1 — Additional file 1. [file 12940_2022_851_MOESM1_ESM.docx]

## Additional file 1. Directed acyclic graph (DAG). Blue: ancestor of outcome. Green: ancestor of exposure. Red: ancestor of outcome and exposure.


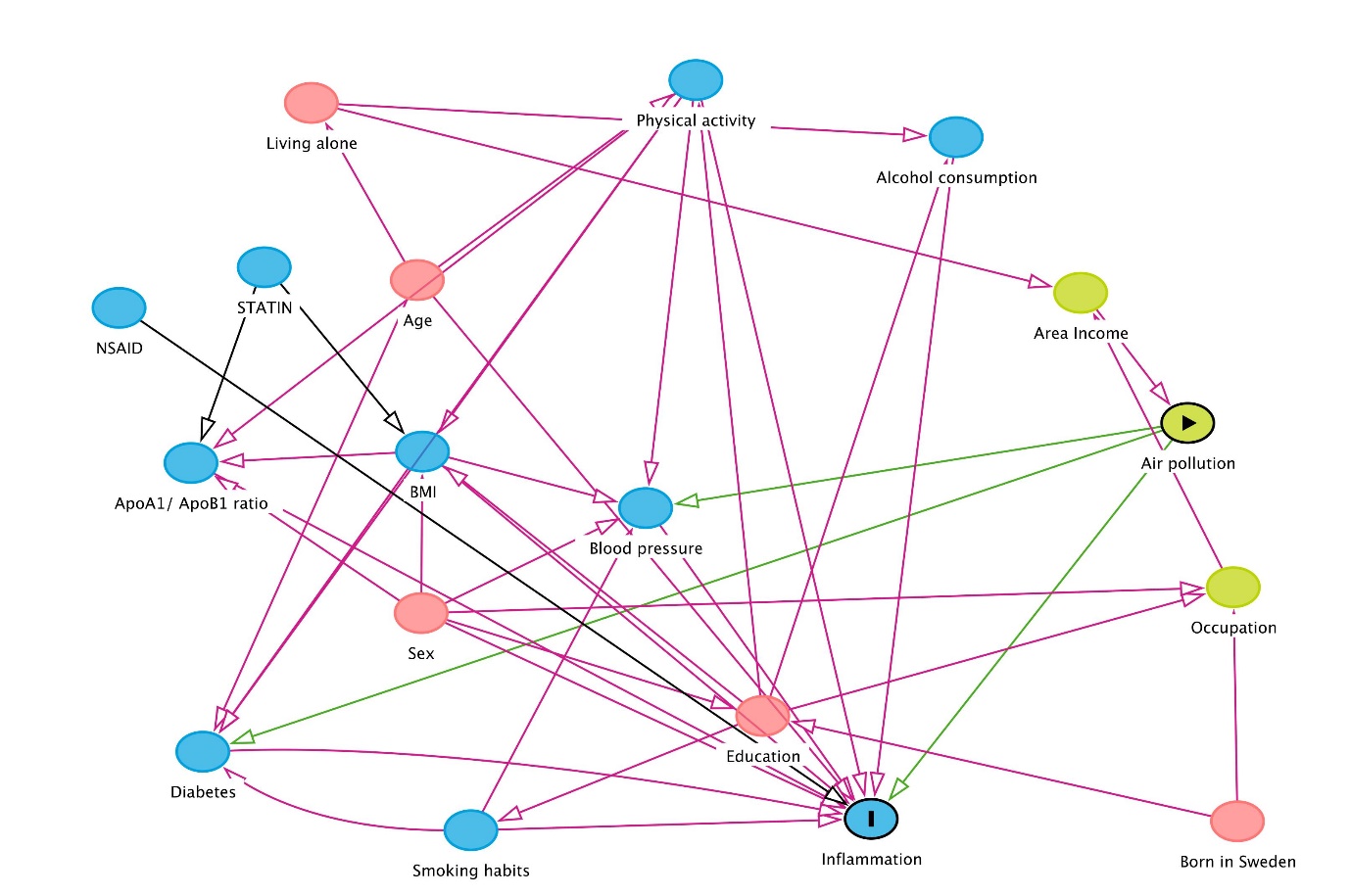


Legend:

*Blue circle with line: outcome*

*green circle with triangle: exposure*

*blue circle: ancestor of outcome*

*green circle: ancestor of exposure*

*pink circle: ancestor of exposure and outcome*

*green arrow: causal path*

*pink arrow: biasing path*
