# Supplementary material for: Air pollution and biomarkers of cardiovascular disease and inflammation in the Malmö Diet and Cancer cohort
Source: Environ Health. 2022 Apr 12;21:39. doi: 10.1186/s12940-022-00851-1 (PMC9004064; doi:10.1186/s12940-022-00851-1)
Supplement: Supplementary file 2 — Additional file 2. [file 12940_2022_851_MOESM2_ESM.docx]

## Additional file 2. Exposure levels of population (μg/m³)­.

| Air pollution (μg/m³)­ | All (n=6057) | Q1 (range) | Q2 (range) | Q3 (range) | Q4 (range) | Missing (n) |
| --- | --- | --- | --- | --- | --- | --- |
| Total PM_10_ (mean, p5-p95) | 14.86 (12.15-17.49) | 10.12 - 13.74 | 13.74 - 14.91 | 14.91 - 15.95 | 15.95 - 24.73 | 45 |
| Total PM_2.5_ (mean, p5-p95) | 10.46 (8.61 – 11.37) | 7.79 -10.19 | 10.19 - 10.55 | 10.55 - 10.99 | 10.99 - 12.68 | 45 |
| Total PM coarse (mean, p5-p95) | 4.41 (2.78 – 6.30) | 2.3 - 3.58 | 3.58 - 4.33 | 4.33 - 5.07 | 5.07 - 12.75 | 45 |
| Total NO_x_ (mean, p5-p95) | 39.1 (18.72 – 62.72) | 12.55 - 29.45 | 29.45 - 37.61 | 37.61 - 47.58 | 47.60 - 127.63 | 45 |
| PM_10_ traffic (mean, p5-p95) | 2.81 (0.81 – 5.11) | .345 - 1.82 | 1.82 - 2.72 | 2.72 - 3.65 | 3.65 - 11.78 | 45 |
| PM_10_ non-traffic (mean, p5-p95) | 0.87 (0.55 – 1.07) | .28 - .77 | .77 - .92 | .92 - .99 | .99 - 1.99 | 45 |
