# Supplementary material for: Air pollution and biomarkers of cardiovascular disease and inflammation in the Malmö Diet and Cancer cohort
Source: Environ Health. 2022 Apr 12;21:39. doi: 10.1186/s12940-022-00851-1 (PMC9004064; doi:10.1186/s12940-022-00851-1)
Supplement: Supplementary file 3 — Additional file 3. [file 12940_2022_851_MOESM3_ESM.docx]

## Additional file 3. Spearman correlation coefficients between different of exposure metrics. P-values for all coefficients are <0.001

|  | PM_10_ | PM_2.5_ | PM coarse | NO_x_ | PM_10_ traffic | PM_10_ non-traffic |
| --- | --- | --- | --- | --- | --- | --- |
| PM_10_ | 1 |  |  |  |  |  |
| PM_2.5_ | 0.88 | 1 |  |  |  |  |
| PM coarse | 0.94 | 0.69 | 1 |  |  |  |
| NO_x_ | 0.88 | 0.59 | 0.97 | 1 |  |  |
| PM_10_ traffic | 0.89 | 0.59 | 0.99 | 0.99 | 1 |  |
| PM_10_ non-traffic | 0.76 | 0.57 | 0.74 | 0.81 | 0.76 | 1 |
