# Supplementary material for: Air pollution and biomarkers of cardiovascular disease and inflammation in the Malmö Diet and Cancer cohort
Source: Environ Health. 2022 Apr 12;21:39. doi: 10.1186/s12940-022-00851-1 (PMC9004064; doi:10.1186/s12940-022-00851-1)
Supplement: Supplementary file 4 — Additional file 4. [file 12940_2022_851_MOESM4_ESM.docx]

## Additional file 4. Spearman correlation between outcomes. P-values for all coefficients are <0.001

|  | CRP | Leukocyte count | NLR | suPAR | Lp-PLA_2_ | Ceruloplasmin | Orosomucoid | Haptoglobin | C3 | Alfa1-antitrypsin |
| --- | --- | --- | --- | --- | --- | --- | --- | --- | --- | --- |
| CRP | 1 |  |  |  |  |  |  |  |  |  |
| Leukocyte count | 0.27 | 1 |  |  |  |  |  |  |  |  |
| NLR | 0.09 | 0.29 | 1 |  |  |  |  |  |  |  |
| suPAR | 0.25 | 0.27 | 0.09 | 1 |  |  |  |  |  |  |
| Lp—PLA_2_ | 0.1 | 0.1 | 0.09 | 0.16 | 1 |  |  |  |  |  |
| Ceruloplasmin | 0.37 | 0.11 | 0.02 | 0.16 | -0.05 | 1 |  |  |  |  |
| Orosomucoid | 0.5 | 0.2 | 0.1 | 0.23 | 0.11 | 0.46 | 1 |  |  |  |
| Haptoglobin | 0.43 | 0.27 | 0.09 | 0.2 | 0.06 | 0.41 | 0.52 | 1 |  |  |
| C3 | 0.44 | 0.17 | -0.00001 | 0.12 | 0.06 | 0.47 | 0.57 | 0.46 | 1 |  |
| Alfa1-antitrypsin | 0.26 | 0.16 | 0.11 | 0.19 | 0.09 | 0.47 | 0.34 | 0.35 | 0.32 | 1 |
