# Supplementary material for: Air pollution and biomarkers of cardiovascular disease and inflammation in the Malmö Diet and Cancer cohort
Source: Environ Health. 2022 Apr 12;21:39. doi: 10.1186/s12940-022-00851-1 (PMC9004064; doi:10.1186/s12940-022-00851-1)
Supplement: Supplementary file 5 — Additional file 5. [file 12940_2022_851_MOESM5_ESM.docx]

## Additional file 5. Sensitivity analyses for the association between PM_2.5_ and biomarkers using the main model (M1): CRP added, season of recruitment added, outliers removed, those with prevalent diabetes removed, those with prevalent CVD removed, as well by using the number of biomarkers above the 75% percentile as the outcome.

|  | CRP |  | Seasons |  | Outliers |  |
| --- | --- | --- | --- | --- | --- | --- |
|  | Added |  | Added |  | Removed |  |
|  | β-Coefficient (95% CI) | P value | β-Coefficient (95% CI) | P value | β-Coefficient (95% CI) | P value |
| Leukocytes | -0.0293 (-0.072 - 0.0133) | 0.178 | -0.0305 (-0.0725 - 0.0115) | 0.155 | -0.0317 (-0.0697 - 0.0063) | 0.102 |
| NLR | 0.0529 (-0.0161 - 0.1218) | 0.133 | **0.0673 (0.0006 - 0.134)** | **0.048** | **0.04 (-0.0179 - 0.098)** | **0.176** |
| CRP |  |  | 0.0249 (-0.1626 - 0.2123) | 0.795 | 0.0238 (-0.1456 - 0.1932) | 0.783 |
| suPAR | -0.0242 (-0.0733 - 0.0249) | 0.333 | -0.0264 (-0.0769 - 0.0241) | 0.305 | -0.0056 (-0.0487 - 0.0374) | 0.797 |
| Lp—PLA_2_ | **0.087 (0.0331 - 0.1409)** | **0.002** | **0.0612 (0.0068 - 0.1155)** | **0.027** | **0.0634 (0.0132 - 0.1137)** | **0.013** |
| Ceruloplasmin | **0.1393 (0.1006 - 0.178)** | **<0.001** | **0.1394 (0.0971 - 0.1816)** | **<0.001** | **0.1139 (0.0777 - 0.1501)** | **<0.001** |
| Orosomucoid | **0.2068 (0.1585 - 0.2551)** | **<0.001** | **0.192 (0.1375 - 0.2465)** | **<0.001** | **0.1632 (0.1143 - 0.2122)** | **<0.001** |
| Haptoglobin | **0.0972 (0.0147 - 0.1796)** | **0.021** | **0.0979 (0.0087 - 0.1871)** | **0.032** | **0.0798 (-0.0039 - 0.1635)** | **0.062** |
| C3 | **0.1707 (0.134 - 0.2074)** | **<0.001** | **0.1476 (0.1087 - 0.1864)** | **<0.001** | **0.1379 (0.1051 - 0.1706)** | **<0.001** |
| Alpha-1-antitrypsin | **0.1143 (0.0682 - 0.1604)** | **<0.001** | **0.0902 (0.0429 - 0.1375)** | **<0.001** | **0.09 (0.0468 - 0.1332)** | **<0.001** |
|  | Prevalent diabetes |  | Prevalent CVD |  |  |  |
|  | Removed |  | Removed |  |  |  |
|  | β-Coefficient (95% CI) |  | β-Coefficient (95% CI) |  |  |  |
| Leukocytes | -0.0096 (-0.0514 - 0.0322) | 0.653 | -0.0153 (-0.0567 - 0.0261) | 0.469 |  |  |
| NLR | **0.0714 (0.0045 - 0.1383)** | **0.036** | 0.0588 (-0.0073 - 0.1248) | 0.081 |  |  |
| CRP | 0.0832 (-0.105 - 0.2714) | 0.386 | 0.0945 (-0.0912 - 0.2802) | 0.319 |  |  |
| suPAR | -0.0065 (-0.057 - 0.044) | 0.8 | -0.0008 (-0.0503 - 0.0486) | 0.973 |  |  |
| Lp—PLA_2_ | **0.1021 (0.0474 - 0.1568)** | **<0.001** | **0.0894 (0.0355 - 0.1432)** | **0.001** |  |  |
| Ceruloplasmin | **0.139 (0.0965 - 0.1815)** | **<0.001** | **0.1392 (0.0973 - 0.1812)** | **<0.001** |  |  |
| Orosomucoid | **0.2163 (0.1615 - 0.2712)** | **<0.001** | **0.2163 (0.1622 - 0.2704)** | **<0.001** |  |  |
| Haptoglobin | **0.1204 (0.0314 - 0.2095)** | **0.008** | **0.1205 (0.0323 - 0.2088)** | **0.007** |  |  |
| C3 | **0.1632 (0.124 - 0.2024)** | **<0.001** | **0.1701 (0.1314 - 0.2089)** | **<0.001** |  |  |
| Alpha-1-antitrypsin | **0.1139 (0.066 - 0.1617)** | **<0.001** | **0.1199 (0.0727 - 0.1671)** | **<0.001** |  |  |
|  | **Biomarkers above 75% percentile*** |  |  |  |  |  |
| Number of biomarkers  (1-10) | **0.11 (0.02 – 0.2)** | **0.022** |  |  |  |  |
|  | **PCA analysis**** |  |  |  |  |  |
|  | β-Coefficient (95% CI) |  |  |  |  |  |
| Component 1 | **0.0252 (0.183 – 0.322)** | **<0.001** |  |  |  |  |
| Component 2 | 0.002 (-0.045 – 0.049) | 0.94 |  |  |  |  |

*Using Poisson regression. **Component 1 includes CRP, ceruloplasmin, orosomucoid, haptoglobin, C3 and alfa-1-antitrypsin while component 2 includes leukocytes, NLR, suPAR and LP-PLA_2_.
