# Supplementary material for: Air pollution and biomarkers of cardiovascular disease and inflammation in the Malmö Diet and Cancer cohort
Source: Environ Health. 2022 Apr 12;21:39. doi: 10.1186/s12940-022-00851-1 (PMC9004064; doi:10.1186/s12940-022-00851-1)
Supplement: Supplementary file 6 — Additional file 6. [file 12940_2022_851_MOESM6_ESM.docx]

## Additional file 6. Stratified analysis and analysis of interaction for the association between biomarkers and PM_2.5_ using the main model (M1). Stratified by age, sex and smoking status.

|  | Sex |  |  |  |  |
| --- | --- | --- | --- | --- | --- |
|  | Men |  | Women |  |  |
|  | β-Coefficient (95% CI) | P value | β-Coefficient (95% CI) | P value | P value (interaction) |
| Leukocytes | -0.0297 (-0.0909 - 0.0315) | 0.341 | -0.0129 (-0.0686 - 0.0427) | 0.649 | 0.683 |
| NLR | **0.1249 (0.026 - 0.2238)** | **0.013** | 0.0221 (-0.0649 - 0.1092) | 0.618 | 0.149 |
| CRP | 0.0913 (-0.1927 - 0.3753) | 0.528 | 0.0288 (-0.2102 - 0.2677) | 0.813 | 0.696 |
| suPAR | -0.0229 (-0.1065 - 0.0606) | 0.59 | -0.0131 (-0.0739 - 0.0477) | 0.672 | 0.822 |
| Lp—PLA_2_ | **0.1267 (0.0457 - 0.2077)** | **0.002** | 0.0531 (-0.0182 - 0.1244) | 0.144 | 0.165 |
| Ceruloplasmin | **0.0773 (0.0147 - 0.1399)** | **0.016** | **0.1757 (0.1207 - 0.2307)** | **<0.001** | **0.035** |
| Orosomucoid | **0.1203 (0.0368 - 0.2037)** | **0.005** | **0.2602 (0.191 - 0.3294)** | **<0.001** | **0.037** |
| Haptoglobin | 0.0974 (-0.0519 - 0.2467) | 0.201 | **0.1095 (0.0037 - 0.2154)** | **0.042** | 0.924 |
| C3 | **0.1265 (0.0685 - 0.1845)** | **<0.001** | **0.1962 (0.1452 - 0.2472)** | **<0.001** | 0.106 |
| Alpha-1-antitrypsin | **0.0738 (0.0016 - 0.1461)** | **0.045** | **0.1411 (0.0802 - 0.202)** | **<0.001** | 0.141 |
|  |  |  |  |  |  |
|  | Age |  |  |  |  |
|  | Young |  | Old |  |  |
|  | β-Coefficient (95% CI) | P value | β-Coefficient (95% CI) | P value | P value (interaction) |
| Leukocytes | 0.0138 (-0.0474 - 0.0751) | 0.658 | -0.047 (-0.1027 - 0.0088) | 0.099 | 0.089 |
| NLR | 0.0807 (-0.0139 - 0.1752) | 0.094 | 0.0628 (-0.0277 - 0.1533) | 0.174 | 0.576 |
| CRP | 0.1551 (-0.1145 - 0.4247) | 0.259 | -0.0055 (-0.2568 - 0.2457) | 0.966 | 0.304 |
| suPAR | -0.0341 (-0.1089 - 0.0407) | 0.371 | -0.0007 (-0.0668 - 0.0653) | 0.982 | 0.769 |
| Lp—PLA_2_ | 0.038 (-0.0415 - 0.1174) | 0.348 | **0.1245 (0.052 - 0.1969)** | **0.001** | 0.143 |
| Ceruloplasmin | **0.1405 (0.0758 - 0.2052)** | **<0.001** | **0.1325 (0.0789 - 0.1861)** | **<0.001** | 0.893 |
| Orosomucoid | **0.2029 (0.1236 - 0.2822)** | **<0.001** | **0.2032 (0.1303 - 0.2761)** | **<0.001** | 0.829 |
| Haptoglobin | **0.196 (0.0622 - 0.3298)** | **0.004** | 0.0337 (-0.082 - 0.1494) | 0.568 | 0.079 |
| C3 | **0.1707 (0.1121 - 0.2294)** | **<0.001** | **0.1651 (0.1145 - 0.2158)** | **<0.001** | 0.983 |
| Alpha-1-antitrypsin | **0.14 (0.0711 - 0.2089)** | **<0.001** | **0.0972 (0.0338 - 0.1606)** | **0.003** | 0.313 |
|  |  |  |  |  |  |
|  | Smoking status |  |  |  |  |
|  | Never-smoker |  | Smoker |  |  |
|  | β-Coefficient (95% CI) | P value | β-Coefficient (95% CI) | P value | P value (interaction) |
| Leukocytes | -0.0157 (-0.0805 - 0.0491) | 0.634 | -0.0182 (-0.0712 - 0.0348) | 0.501 | 0.781 |
| NLR | -0.0092 (-0.1131 - 0.0947) | 0.863 | **0.1138 (0.0294 - 0.1983)** | **0.008** | 0.098 |
| CRP | 0.1907 (-0.0886 - 0.47) | 0.181 | -0.0224 (-0.2648 - 0.22) | 0.856 | 0.102 |
| suPAR | 0.0209 (-0.0474 - 0.0892) | 0.549 | -0.0374 (-0.1059 - 0.031) | 0.284 | 0.151 |
| Lp—PLA_2_ | **0.0869 (0.0016 - 0.1723)** | **0.046** | **0.0854 (0.0167 - 0.1542)** | **0.015** | 0.888 |
| Ceruloplasmin | **0.1885 (0.1243 - 0.2527)** | **<0.001** | **0.1003 (0.046 - 0.1546)** | **<0.001** | **0.022** |
| Orosomucoid | **0.2511 (0.169 - 0.3333)** | **<0.001** | **0.1712 (0.1007 - 0.2416)** | **<0.001** | 0.130 |
| Haptoglobin | 0.0591 (-0.0804 - 0.1985) | 0.406 | **0.1395 (0.0275 - 0.2515)** | **0.015** | 0.494 |
| C3 | **0.1823 (0.1217 - 0.2429)** | **<0.001** | **0.1559 (0.1063 - 0.2055)** | **<0.001** | 0.431 |
| Alpha-1-antitrypsin | **0.1842 (0.1112 - 0.2572)** | **<0.001** | **0.0689 (0.0085 - 0.1293)** | **0.025** | **0.007** |
