# Supplementary material for: Air pollution and biomarkers of cardiovascular disease and inflammation in the Malmö Diet and Cancer cohort
Source: Environ Health. 2022 Apr 12;21:39. doi: 10.1186/s12940-022-00851-1 (PMC9004064; doi:10.1186/s12940-022-00851-1)
Supplement: Supplementary file 7 — Additional file 7. [file 12940_2022_851_MOESM7_ESM.docx]

## Additional file 7. Quartile analysis for the association between biomarkers and air pollution exposure.

| PM_2.5_ |  |  |  |  |  |  |  |
| --- | --- | --- | --- | --- | --- | --- | --- |
|  |  | Q2: β-Coefficient (95% CI) | P-value | Q3: β-Coefficient (95% CI) | P-value | Q4: β-Coefficient (95% CI) | P-value |
| Leukocyte count | M0 | -0.042 (-0.13 - 0.05) | 0.369 | -0.081 (-0.17 - 0.01) | 0.081 | -0.0067 (-0.098 - 0.085) | 0.886 |
|  | M1 | -0.023 (-0.11 - 0.064) | 0.602 | -0.075 (-0.16 - 0.012) | 0.091 | -0.018 (-0.1 - 0.07) | 0.695 |
|  | M2 | 0.0001 (-0.088 - 0.088) | 0.999 | -0.065 (-0.15 - 0.023) | 0.148 | -0.031 (-0.12 - 0.059) | 0.496 |
| NLR | M0 | 0.0264 (-0.1087 - 0.1616) | 0.701 | 0.031 (-0.1 - 0.17) | 0.654 | **0.16 (0.025 - 0.3)** | **0.02** |
|  | M1 | 0.0164 (-0.1216 - 0.1544) | 0.816 | 0.0158 (-0.1222 - 0.1537) | 0.823 | 0.1259 (-0.0127 - 0.2645) | 0.075 |
|  | M2 | -0.0008 (-0.1432 - 0.1416) | 0.991 | 0.0259 (-0.1173 - 0.1691) | 0.723 | 0.1421 (-0.0041 - 0.2883) | 0.057 |
| CRP | M0 | -0.3583 (-0.7628 - 0.0462) | 0.083 | **-0.4529 (-0.8565 - -0.0492)** | **0.028** | -0.1751 (-0.5828 - 0.2327) | 0.4 |
|  | M1 | -0.2665 (-0.6522 - 0.1193) | 0.176 | -0.3641 (-0.749 - 0.0208) | 0.064 | -0.0355 (-0.4249 - 0.354) | 0.858 |
|  | M2 | -0.2981 (-0.6904 - 0.0943) | 0.136 | -0.3707 (-0.765 - 0.0236) | 0.065 | -0.1236 (-0.5296 - 0.2823) | 0.55 |
| Lp—PLA_2_ | M0 | 0.0365 (-0.0746 - 0.1476) | 0.519 | 0.0776 (-0.0334 - 0.1886) | 0.171 | 0.1094 (-0.0027 - 0.2214) | 0.056 |
|  | M1 | 0.0219 (-0.0899 - 0.1337) | 0.701 | 0.0888 (-0.0229 - 0.2005) | 0.119 | 0.0843 (-0.0287 - 0.1973) | 0.144 |
|  | M2 | 0.0413 (-0.0713 - 0.154) | 0.472 | 0.1098 (-0.0035 - 0.2231) | 0.057 | 0.0852 (-0.0314 - 0.2019) | 0.152 |
| Ceruloplasmin | M0 | **0.0936 (0.0084 - 0.1789)** | **0.031** | **0.0985 (0.0134 - 0.1836)** | **0.023** | **0.2074 (0.1213 - 0.2936)** | **<0.001** |
|  | M1 | **0.0969 (0.011 - 0.1828)** | **0.027** | **0.1025 (0.0167 - 0.1883)** | **0.019** | **0.2082 (0.1212 - 0.2952)** | **<0.001** |
|  | M2 | 0.087 (-0.0004 - 0.1744) | 0.051 | **0.1058 (0.0177 - 0.1939)** | **0.019** | **0.1838 (0.0929 - 0.2748)** | **<0.001** |
| Orosomucoid | M0 | 0.1116 (-0.001 - 0.2243) | 0.052 | **0.194 (0.0815 - 0.3065)** | **0.001** | **0.3423 (0.2286 - 0.456)** | **<0.001** |
|  | M1 | **0.1249 (0.0148 - 0.2349)** | **0.026** | **0.2203 (0.1104 - 0.3302)** | **<0.001** | **0.3605 (0.2493 - 0.4718)** | **<0.001** |
|  | M2 | **0.1296 (0.0194 - 0.2398)** | 0.021 | **0.2287 (0.1178 - 0.3396)** | <0.001 | **0.3493 (0.235 - 0.4636)** | **<0.001** |
| Haptoglobin | M0 | 0.0127 (-0.1735 - 0.1989) | 0.894 | 0.0788 (-0.1069 - 0.2644) | 0.406 | 0.1493 (-0.0382 - 0.3367) | 0.119 |
|  | M1 | 0.0122 (-0.1701 - 0.1944) | 0.896 | 0.0829 (-0.0991 - 0.2648) | 0.372 | 0.1324 (-0.0515 - 0.3164) | 0.158 |
|  | M2 | -0.0032 (-0.1882 - 0.1819) | 0.973 | 0.0683 (-0.118 - 0.2547) | 0.472 | 0.0712 (-0.1206 - 0.263) | 0.467 |
| C3 | M0 | **0.0967 (0.0125 - 0.1809)** | **0.024** | **0.1381 (0.0541 - 0.2222)** | **0.001** | **0.216 (0.131 - 0.301)** | **<0.001** |
|  | M1 | **0.1173 (0.0385 - 0.196)** | **0.004** | **0.1674 (0.0887 - 0.2461)** | **<0.001** | **0.2612 (0.1815 - 0.3408)** | **<0.001** |
|  | M2 | **0.1208 (0.0421 - 0.1995)** | **0.003** | **0.1618 (0.0826 - 0.241)** | **<0.001** | **0.2347 (0.1531 - 0.3164)** | **<0.001** |
| alfa-1-antitrypsin | M0 | 0.014 (-0.0811 - 0.1091) | 0.773 | 0.0754 (-0.0196 - 0.1703) | 0.12 | 0.1914 (0.0954 - 0.2874) | <0.001 |
|  | M1 | 0.0001 (-0.0957 - 0.0959) | 0.998 | 0.075 (-0.0207 - 0.1706) | 0.124 | 0.1663 (0.0694 - 0.2631) | 0.001 |
|  | M2 | 0.0121 (-0.0863 - 0.1105) | 0.809 | 0.0781 (-0.0211 - 0.1772) | 0.123 | 0.1393 (0.0372 - 0.2415) | 0.008 |
| suPAR | M0 | -0.0392 (-0.1483 - 0.0699) | 0.481 | -0.0225 (-0.1314 - 0.0863) | 0.685 | 0.062 (-0.0475 - 0.1715) | 0.267 |
|  | M1 | -0.0256 (-0.1286 - 0.0775) | 0.626 | -0.0002 (-0.1029 - 0.1026) | 0.998 | 0.0516 (-0.052 - 0.1553) | 0.329 |
|  | M2 | 0.0145 (-0.0888 - 0.1177) | 0.783 | -0.0188 (-0.1224 - 0.0848) | 0.722 | 0.0428 (-0.0637 - 0.1492) | 0.431 |
| PM_10_ |  |  |  |  |  |  |  |
|  |  | Q2: β-Coefficient (95% CI) | P-value | Q3: β-Coefficient (95% CI) | P-value | Q4: β-Coefficient (95% CI) |  |
| Leukocyte count | M0 | -0.0832 (-0.1747 - 0.0082) | 0.074 | 0.0134 (-0.0782 - 0.105) | 0.774 | 0.0421 (-0.0494 - 0.1336) | 0.367 |
|  | M1 | -0.0687 (-0.1554 - 0.018) | 0.121 | -0.0127 (-0.0999 - 0.0745) | 0.775 | -0.0228 (-0.1104 - 0.0648) | 0.61 |
|  | M2 | -0.0536 (-0.1409 - 0.0338) | 0.229 | -0.0262 (-0.1169 - 0.0645) | 0.571 | -0.0531 (-0.147 - 0.0408) | 0.267 |
| NLR | M0 | 0.0214 (-0.1138 - 0.1567) | 0.756 | -0.0185 (-0.1539 - 0.1168) | 0.788 | 0.1287 (-0.0066 - 0.2639) | 0.062 |
|  | M1 | 0.0181 (-0.1196 - 0.1557) | 0.797 | -0.042 (-0.1804 - 0.0963) | 0.552 | 0.105 (-0.034 - 0.2441) | 0.139 |
|  | M2 | 0.0296 (-0.1121 - 0.1713) | 0.682 | -0.0245 (-0.1716 - 0.1227) | 0.745 | 0.1329 (-0.0194 - 0.2852) | 0.087 |
| CRP | M0 | -0.435 (-0.8371 - -0.0328) | 0.034 | -0.1772 (-0.58 - 0.2257) | 0.389 | 0.2081 (-0.1989 - 0.6151) | 0.316 |
|  | M1 | -0.1773 (-0.5603 - 0.2057) | 0.364 | -0.119 (-0.5036 - 0.2657) | 0.544 | 0.1713 (-0.2185 - 0.561) | 0.389 |
|  | M2 | -0.1781 (-0.5673 - 0.2111) | 0.37 | -0.2496 (-0.6534 - 0.1543) | 0.226 | 0.082 (-0.3386 - 0.5026) | 0.702 |
| Lp—PLA_2_ | M0 | 0.1204 (0.0096 - 0.2312) | 0.033 | 0.1402 (0.029 - 0.2514) | 0.013 | 0.0914 (-0.0207 - 0.2036) | 0.11 |
|  | M1 | 0.1194 (0.0081 - 0.2307) | 0.035 | 0.1255 (0.0135 - 0.2374) | 0.028 | 0.0523 (-0.0609 - 0.1656) | 0.365 |
|  | M2 | 0.1071 (-0.0049 - 0.2191) | 0.061 | 0.1541 (0.0376 - 0.2706) | 0.01 | 0.0784 (-0.0428 - 0.1996) | 0.205 |
| Ceruloplasmin | M0 | 0.0973 (0.0126 - 0.182) | 0.024 | 0.1568 (0.0718 - 0.2418) | <0.001 | 0.1802 (0.0944 - 0.2659) | <0.001 |
|  | M1 | 0.0971 (0.0119 - 0.1823) | 0.026 | 0.1467 (0.0608 - 0.2325) | 0.001 | 0.1744 (0.0876 - 0.2612) | <0.001 |
|  | M2 | 0.0964 (0.0098 - 0.1831) | 0.029 | 0.1288 (0.0383 - 0.2193) | 0.005 | 0.1636 (0.0696 - 0.2577) | 0.001 |
| Orosomucoid | M0 | 0.123 (0.0103 - 0.2358) | 0.032 | 0.1915 (0.0789 - 0.3041) | 0.001 | 0.2652 (0.1514 - 0.3791) | <0.001 |
|  | M1 | 0.1681 (0.0582 - 0.2781) | 0.003 | 0.2074 (0.0973 - 0.3176) | <0.001 | 0.2526 (0.1409 - 0.3644) | <0.001 |
|  | M2 | 0.1653 (0.0554 - 0.2753) | 0.003 | 0.2048 (0.0905 - 0.319) | <0.001 | 0.2512 (0.1322 - 0.3702) | <0.001 |
| Haptoglobin | M0 | 0.0512 (-0.1338 - 0.2362) | 0.588 | 0.1548 (-0.0302 - 0.3398) | 0.101 | 0.2155 (0.0285 - 0.4025) | 0.024 |
|  | M1 | 0.0731 (-0.1077 - 0.2539) | 0.428 | 0.1103 (-0.0712 - 0.2918) | 0.234 | 0.1645 (-0.0194 - 0.3483) | 0.08 |
|  | M2 | 0.0802 (-0.1034 - 0.2637) | 0.392 | 0.0527 (-0.138 - 0.2435) | 0.588 | 0.1245 (-0.0741 - 0.3232) | 0.219 |
| C3 | M0 | 0.0904 (0.0063 - 0.1746) | 0.035 | 0.1271 (0.0429 - 0.2112) | 0.003 | 0.1864 (0.1014 - 0.2714) | <0.001 |
|  | M1 | 0.1335 (0.0549 - 0.2121) | 0.001 | 0.1564 (0.0775 - 0.2353) | <0.001 | 0.1969 (0.117 - 0.2768) | <0.001 |
|  | M2 | 0.1302 (0.0517 - 0.2087) | 0.001 | 0.1458 (0.0641 - 0.2274) | <0.001 | 0.1774 (0.0925 - 0.2623) | <0.001 |
| alfa-1-antitrypsin | M0 | 0.0463 (-0.0486 - 0.1411) | 0.339 | 0.1618 (0.0669 - 0.2567) | 0.001 | 0.1722 (0.0763 - 0.268) | <0.001 |
|  | M1 | 0.0363 (-0.059 - 0.1317) | 0.455 | 0.1327 (0.037 - 0.2285) | 0.007 | 0.1501 (0.0532 - 0.2471) | 0.002 |
|  | M2 | 0.0446 (-0.0533 - 0.1426) | 0.371 | 0.11 (0.0081 - 0.2119) | 0.034 | 0.1223 (0.0162 - 0.2284) | 0.024 |
| suPAR | M0 | -0.0303 (-0.139 - 0.0784) | 0.585 | 0.0006 (-0.1083 - 0.1096) | 0.991 | 0.1175 (0.0077 - 0.2273) | 0.036 |
|  | M1 | -0.0003 (-0.1028 - 0.1021) | 0.995 | -0.0345 (-0.1376 - 0.0685) | 0.511 | 0.0547 (-0.0495 - 0.1588) | 0.304 |
|  | M2 | 0.0083 (-0.0943 - 0.1108) | 0.874 | -0.0614 (-0.168 - 0.0452) | 0.259 | 0.0168 (-0.0941 - 0.1277) | 0.767 |
| NO_x_ |  |  |  |  |  |  |  |
|  |  | Q2: β-Coefficient (95% CI) | P-value | Q3: β-Coefficient (95% CI) | P-value | Q4: β-Coefficient (95% CI) |  |
| Leukocyte count | M0 | -0.0301 (-0.3965 - 0.3363) | 0.872 | 0.3439 (-0.0236 - 0.7114) | 0.067 | 0.4396 (0.0736 - 0.8057) | 0.019 |
|  | M1 | -0.0841 (-0.4308 - 0.2625) | 0.634 | 0.0691 (-0.2809 - 0.4191) | 0.699 | 0.0465 (-0.3039 - 0.3969) | 0.795 |
|  | M2 | -0.0877 (-0.4381 - 0.2627) | 0.624 | -0.0148 (-0.3889 - 0.3594) | 0.938 | -0.0696 (-0.4564 - 0.3172) | 0.724 |
| NLR | M0 | -0.3089 (-0.8508 - 0.233) | 0.264 | -0.1187 (-0.6624 - 0.4249) | 0.669 | 0.0312 (-0.5106 - 0.573) | 0.91 |
|  | M1 | -0.3395 (-0.8898 - 0.2107) | 0.226 | -0.3488 (-0.9045 - 0.2068) | 0.218 | -0.0563 (-0.6128 - 0.5002) | 0.843 |
|  | M2 | -0.3329 (-0.9013 - 0.2356) | 0.251 | -0.427 (-1.0338 - 0.1799) | 0.168 | -0.0344 (-0.6618 - 0.593) | 0.914 |
| CRP | M0 | -0.9077 (-2.5142 - 0.6988) | 0.268 | 0.6048 (-1.0139 - 2.2234) | 0.464 | 0.694 (-0.9306 - 2.3186) | 0.402 |
|  | M1 | -0.6377 (-2.1656 - 0.8903) | 0.413 | 0.2427 (-1.3024 - 1.7878) | 0.758 | 0.0654 (-1.4898 - 1.6206) | 0.934 |
|  | M2 | -0.9235 (-2.4855 - 0.6385) | 0.246 | -0.2463 (-1.911 - 1.4184) | 0.772 | -0.6609 (-2.3917 - 1.0699) | 0.454 |
| Lp—PLA_2_ | M0 | 0.3566 (-0.0866 - 0.7999) | 0.115 | 0.3394 (-0.1072 - 0.786) | 0.136 | 0.0712 (-0.3756 - 0.518) | 0.755 |
|  | M1 | 0.3129 (-0.1318 - 0.7577) | 0.168 | 0.169 (-0.2807 - 0.6188) | 0.461 | -0.1073 (-0.5586 - 0.344) | 0.641 |
|  | M2 | 0.4642 (0.0142 - 0.9142) | 0.043 | 0.3597 (-0.12 - 0.8395) | 0.142 | 0.1395 (-0.3584 - 0.6374) | 0.583 |
| Ceruloplasmin | M0 | -0.084 (-0.4227 - 0.2547) | 0.627 | 0.0908 (-0.2495 - 0.431) | 0.601 | 0.2452 (-0.0963 - 0.5867) | 0.159 |
|  | M1 | -0.1037 (-0.4438 - 0.2364) | 0.55 | 0.0569 (-0.2863 - 0.4002) | 0.745 | 0.2159 (-0.13 - 0.5617) | 0.221 |
|  | M2 | -0.1832 (-0.5316 - 0.1651) | 0.303 | -0.0835 (-0.4544 - 0.2873) | 0.659 | 0.0504 (-0.3359 - 0.4367) | 0.798 |
| Orosomucoid | M0 | 0.0559 (-0.3945 - 0.5063) | 0.808 | 0.1835 (-0.2685 - 0.6355) | 0.426 | 0.3223 (-0.1301 - 0.7746) | 0.163 |
|  | M1 | 0.065 (-0.3737 - 0.5036) | 0.772 | 0.0983 (-0.3442 - 0.5407) | 0.663 | 0.1936 (-0.2503 - 0.6375) | 0.393 |
|  | M2 | 0.005 (-0.4364 - 0.4463) | 0.982 | -0.0073 (-0.4776 - 0.463) | 0.976 | 0.0472 (-0.4399 - 0.5344) | 0.849 |
| Haptoglobin | M0 | 0.4868 (-0.2502 - 1.2238) | 0.195 | 0.703 (-0.0365 - 1.4425) | 0.062 | 0.7471 (0.0035 - 1.4907) | 0.049 |
|  | M1 | 0.3893 (-0.33 - 1.1087) | 0.289 | 0.4343 (-0.2909 - 1.1595) | 0.24 | 0.3781 (-0.3534 - 1.1097) | 0.311 |
|  | M2 | 0.3514 (-0.3836 - 1.0863) | 0.349 | 0.2133 (-0.5685 - 0.9951) | 0.593 | 0.0691 (-0.7462 - 0.8845) | 0.868 |
| C3 | M0 | 0.0256 (-0.3104 - 0.3617) | 0.881 | -0.0089 (-0.3468 - 0.3291) | 0.959 | 0.2241 (-0.113 - 0.5612) | 0.193 |
|  | M1 | 0.1108 (-0.203 - 0.4246) | 0.489 | 0.0106 (-0.3065 - 0.3277) | 0.948 | 0.2283 (-0.089 - 0.5455) | 0.158 |
|  | M2 | 0.0531 (-0.2617 - 0.368) | 0.741 | -0.1356 (-0.4714 - 0.2002) | 0.429 | 0.0295 (-0.3175 - 0.3765) | 0.868 |
| alfa-1-antitrypsin | M0 | 0.0369 (-0.342 - 0.4159) | 0.848 | 0.1932 (-0.1874 - 0.5737) | 0.32 | 0.3269 (-0.0532 - 0.7071) | 0.092 |
|  | M1 | -0.0494 (-0.4295 - 0.3307) | 0.799 | 0.0425 (-0.3413 - 0.4263) | 0.828 | 0.2195 (-0.165 - 0.604) | 0.263 |
|  | M2 | -0.1311 (-0.5234 - 0.2612) | 0.512 | -0.1775 (-0.596 - 0.241) | 0.406 | -0.0103 (-0.4434 - 0.4229) | 0.963 |
| suPAR | M0 | 0.1984 (-0.2351 - 0.6319) | 0.37 | 0.6947 (0.2587 - 1.1307) | 0.002 | 0.5289 (0.0927 - 0.9652) | 0.017 |
|  | M1 | 0.0686 (-0.3399 - 0.477) | 0.742 | 0.421 (0.0087 - 0.8333) | 0.045 | 0.0976 (-0.3162 - 0.5114) | 0.644 |
|  | M2 | 0.1265 (-0.2844 - 0.5375) | 0.546 | 0.4171 (-0.0202 - 0.8545) | 0.062 | -0.0277 (-0.482 - 0.4265) | 0.905 |
| PM Coarse |  |  |  |  |  |  |  |
|  |  | Q2: β-Coefficient (95% CI) | P-value | Q3: β-Coefficient (95% CI) | P-value | Q4: β-Coefficient (95% CI) |  |
| Leukocyte count | M0 | -0.0117 (-0.1034 - 0.0799) | 0.802 | 0.0829 (-0.0089 - 0.1747) | 0.077 | 0.1228 (0.0314 - 0.2143) | 0.008 |
|  | M1 | -0.0451 (-0.1318 - 0.0416) | 0.308 | 0.0114 (-0.0762 - 0.099) | 0.798 | 0.0236 (-0.0639 - 0.1111) | 0.597 |
|  | M2 | -0.0508 (-0.1394 - 0.0378) | 0.261 | -0.0119 (-0.1065 - 0.0828) | 0.806 | -0.0197 (-0.1168 - 0.0775) | 0.692 |
| NLR | M0 | -0.0581 (-0.1937 - 0.0774) | 0.4 | 0.0037 (-0.1321 - 0.1395) | 0.957 | 0.0312 (-0.1042 - 0.1665) | 0.652 |
|  | M1 | -0.0683 (-0.206 - 0.0694) | 0.331 | -0.047 (-0.1861 - 0.0921) | 0.507 | -0.0015 (-0.1405 - 0.1375) | 0.983 |
|  | M2 | -0.0641 (-0.2078 - 0.0797) | 0.382 | -0.0362 (-0.1897 - 0.1173) | 0.644 | 0.0066 (-0.151 - 0.1643) | 0.934 |
| CRP | M0 | -0.1724 (-0.5738 - 0.2289) | 0.4 | 0.0197 (-0.3852 - 0.4246) | 0.924 | 0.2488 (-0.1578 - 0.6554) | 0.23 |
|  | M1 | -0.2016 (-0.5834 - 0.1802) | 0.301 | -0.0591 (-0.446 - 0.3278) | 0.765 | 0.0232 (-0.3658 - 0.4121) | 0.907 |
|  | M2 | -0.2984 (-0.6922 - 0.0953) | 0.137 | -0.2318 (-0.6529 - 0.1892) | 0.28 | -0.169 (-0.6034 - 0.2655) | 0.446 |
| Lp—PLA_2_ | M0 | 0.0199 (-0.0908 - 0.1307) | 0.724 | 0.0829 (-0.0288 - 0.1947) | 0.146 | 0.0263 (-0.0856 - 0.1382) | 0.645 |
|  | M1 | 0.0055 (-0.1056 - 0.1166) | 0.923 | 0.0416 (-0.0711 - 0.1542) | 0.47 | -0.0236 (-0.1365 - 0.0893) | 0.682 |
|  | M2 | 0.0278 (-0.0857 - 0.1412) | 0.631 | 0.0895 (-0.0319 - 0.2109) | 0.148 | 0.0233 (-0.1017 - 0.1484) | 0.715 |
| Ceruloplasmin | M0 | -0.0022 (-0.0866 - 0.0822) | 0.959 | 0.0898 (0.0045 - 0.175) | 0.039 | 0.1036 (0.0181 - 0.1891) | 0.018 |
|  | M1 | -0.0135 (-0.0983 - 0.0713) | 0.755 | 0.0787 (-0.0074 - 0.1649) | 0.073 | 0.0914 (0.0049 - 0.1779) | 0.038 |
|  | M2 | -0.0356 (-0.1232 - 0.0521) | 0.426 | 0.0469 (-0.0473 - 0.141) | 0.329 | 0.0656 (-0.0313 - 0.1626) | 0.185 |
| Orosomucoid | M0 | 0.036 (-0.0763 - 0.1484) | 0.53 | 0.1649 (0.0518 - 0.278) | 0.004 | 0.1212 (0.008 - 0.2345) | 0.036 |
|  | M1 | 0.0165 (-0.0929 - 0.126) | 0.767 | 0.147 (0.0362 - 0.2578) | 0.009 | 0.0687 (-0.0424 - 0.1797) | 0.225 |
|  | M2 | -0.0014 (-0.1125 - 0.1097) | 0.98 | 0.1225 (0.0036 - 0.2414) | 0.044 | 0.0445 (-0.0778 - 0.1668) | 0.476 |
| Haptoglobin | M0 | 0.1734 (-0.0103 - 0.3571) | 0.064 | 0.257 (0.0715 - 0.4426) | 0.007 | 0.1972 (0.0111 - 0.3833) | 0.038 |
|  | M1 | 0.1335 (-0.0459 - 0.3129) | 0.145 | 0.1795 (-0.0027 - 0.3617) | 0.053 | 0.1117 (-0.0711 - 0.2946) | 0.231 |
|  | M2 | 0.1153 (-0.0695 - 0.3001) | 0.221 | 0.1274 (-0.071 - 0.3258) | 0.208 | 0.0487 (-0.1557 - 0.2531) | 0.641 |
| C3 | M0 | 0.0288 (-0.0551 - 0.1127) | 0.501 | 0.0706 (-0.0139 - 0.1552) | 0.101 | 0.1046 (0.0201 - 0.1891) | 0.015 |
|  | M1 | 0.0305 (-0.0479 - 0.1089) | 0.445 | 0.0802 (0.0009 - 0.1596) | 0.048 | 0.0883 (0.0089 - 0.1678) | 0.029 |
|  | M2 | 0.0188 (-0.0605 - 0.0981) | 0.643 | 0.0482 (-0.0368 - 0.1332) | 0.266 | 0.0504 (-0.0368 - 0.1377) | 0.257 |
| alfa-1-antitrypsin | M0 | 0.0012 (-0.0932 - 0.0957) | 0.979 | 0.1557 (0.0606 - 0.2508) | 0.001 | 0.1197 (0.0245 - 0.215) | 0.014 |
|  | M1 | -0.0256 (-0.1204 - 0.0692) | 0.597 | 0.1204 (0.0243 - 0.2164) | 0.014 | 0.0932 (-0.003 - 0.1895) | 0.058 |
|  | M2 | -0.0355 (-0.1342 - 0.0632) | 0.481 | 0.08 (-0.0259 - 0.186) | 0.139 | 0.0466 (-0.0623 - 0.1555) | 0.402 |
| suPAR | M0 | 0.0733 (-0.035 - 0.1817) | 0.185 | 0.142 (0.0327 - 0.2512) | 0.011 | 0.1838 (0.0745 - 0.293) | 0.001 |
|  | M1 | 0.0369 (-0.0652 - 0.1391) | 0.478 | 0.0664 (-0.037 - 0.1699) | 0.208 | 0.0688 (-0.0347 - 0.1724) | 0.193 |
|  | M2 | 0.0368 (-0.0669 - 0.1404) | 0.487 | 0.05 (-0.0607 - 0.1607) | 0.376 | 0.0265 (-0.0876 - 0.1406) | 0.649 |
| PM_10_ traffic |  |  |  |  |  |  |  |
|  |  | Q2: β-Coefficient (95% CI) | P-value | Q3: β-Coefficient (95% CI) | P-value | Q4: β-Coefficient (95% CI) |  |
| Leukocyte count | M0 | 0.0004 (-0.018 - 0.0187) | 0.97 | 0.0198 (0.0014 - 0.0381) | 0.035 | 0.0223 (0.004 - 0.0406) | 0.017 |
|  | M1 | -0.0047 (-0.0221 - 0.0126) | 0.592 | 0.0063 (-0.0112 - 0.0239) | 0.479 | 0.0014 (-0.0161 - 0.0189) | 0.877 |
|  | M2 | -0.0055 (-0.0233 - 0.0122) | 0.541 | 0.0022 (-0.0169 - 0.0214) | 0.819 | -0.006 (-0.0256 - 0.0136) | 0.55 |
| NLR | M0 | -0.0187 (-0.0458 - 0.0084) | 0.175 | 0.0031 (-0.0241 - 0.0303) | 0.824 | 0.0004 (-0.0267 - 0.0274) | 0.979 |
|  | M1 | -0.0223 (-0.0498 - 0.0053) | 0.113 | -0.0083 (-0.0362 - 0.0195) | 0.559 | -0.0049 (-0.0327 - 0.0229) | 0.731 |
|  | M2 | -0.0196 (-0.0485 - 0.0092) | 0.182 | -0.0086 (-0.0396 - 0.0225) | 0.589 | -0.002 (-0.0338 - 0.0298) | 0.903 |
| CRP | M0 | -0.0236 (-0.1039 - 0.0568) | 0.565 | 0.0393 (-0.0418 - 0.1204) | 0.342 | 0.0489 (-0.0324 - 0.1301) | 0.238 |
|  | M1 | -0.012 (-0.0884 - 0.0644) | 0.758 | 0.0162 (-0.0613 - 0.0938) | 0.682 | 0.0099 (-0.0679 - 0.0877) | 0.803 |
|  | M2 | -0.0314 (-0.1105 - 0.0478) | 0.437 | -0.0135 (-0.0987 - 0.0718) | 0.757 | -0.0284 (-0.116 - 0.0593) | 0.526 |
| Lp—PLA_2_ | M0 | 0.0038 (-0.0184 - 0.0259) | 0.738 | 0.0123 (-0.0101 - 0.0347) | 0.282 | -0.0004 (-0.0228 - 0.0219) | 0.971 |
|  | M1 | -0.0002 (-0.0225 - 0.022) | 0.984 | 0.0046 (-0.018 - 0.0271) | 0.692 | -0.0105 (-0.0331 - 0.0121) | 0.363 |
|  | M2 | 0.0086 (-0.0142 - 0.0314) | 0.457 | 0.0145 (-0.01 - 0.0391) | 0.246 | 0.0016 (-0.0236 - 0.0268) | 0.902 |
| Ceruloplasmin | M0 | 0.001 (-0.0159 - 0.018) | 0.905 | 0.0074 (-0.0097 - 0.0244) | 0.398 | 0.0135 (-0.0036 - 0.0306) | 0.122 |
|  | M1 | -0.0005 (-0.0175 - 0.0165) | 0.951 | 0.0054 (-0.0119 - 0.0226) | 0.543 | 0.0117 (-0.0057 - 0.029) | 0.187 |
|  | M2 | -0.0069 (-0.0245 - 0.0107) | 0.444 | -0.003 (-0.022 - 0.016) | 0.756 | 0.0027 (-0.0168 - 0.0223) | 0.784 |
| Orosomucoid | M0 | 0.0057 (-0.0168 - 0.0283) | 0.617 | 0.0218 (-0.0008 - 0.0445) | 0.059 | 0.0133 (-0.0093 - 0.0359) | 0.249 |
|  | M1 | 0.005 (-0.0169 - 0.0269) | 0.655 | 0.0161 (-0.0061 - 0.0383) | 0.156 | 0.0058 (-0.0164 - 0.028) | 0.611 |
|  | M2 | 0.0016 (-0.0208 - 0.0239) | 0.889 | 0.0105 (-0.0135 - 0.0346) | 0.391 | -0.0014 (-0.0261 - 0.0232) | 0.908 |
| Haptoglobin | M0 | 0.0358 (-0.0011 - 0.0726) | 0.057 | 0.0438 (0.0068 - 0.0809) | 0.021 | 0.0407 (0.0035 - 0.0778) | 0.032 |
|  | M1 | 0.0301 (-0.0059 - 0.0661) | 0.101 | 0.0297 (-0.0068 - 0.0661) | 0.11 | 0.0226 (-0.014 - 0.0591) | 0.226 |
|  | M2 | 0.0233 (-0.0139 - 0.0606) | 0.219 | 0.0176 (-0.0225 - 0.0577) | 0.389 | 0.0074 (-0.0339 - 0.0486) | 0.727 |
| C3 | M0 | 0.0054 (-0.0114 - 0.0222) | 0.528 | 0.0062 (-0.0107 - 0.0231) | 0.472 | 0.0112 (-0.0057 - 0.0281) | 0.193 |
|  | M1 | 0.0098 (-0.0059 - 0.0255) | 0.221 | 0.0047 (-0.0112 - 0.0206) | 0.565 | 0.0109 (-0.005 - 0.0268) | 0.179 |
|  | M2 | 0.0054 (-0.0106 - 0.0213) | 0.508 | -0.0031 (-0.0203 - 0.0141) | 0.725 | 0.0009 (-0.0167 - 0.0185) | 0.919 |
| alfa-1-antitrypsin | M0 | 0.0115 (-0.0074 - 0.0305) | 0.233 | 0.0158 (-0.0033 - 0.0348) | 0.105 | 0.0223 (0.0032 - 0.0413) | 0.022 |
|  | M1 | 0.0064 (-0.0126 - 0.0254) | 0.509 | 0.0078 (-0.0114 - 0.0271) | 0.425 | 0.0174 (-0.0018 - 0.0367) | 0.076 |
|  | M2 | 0.0026 (-0.0173 - 0.0224) | 0.799 | -0.0027 (-0.0241 - 0.0187) | 0.803 | 0.0063 (-0.0156 - 0.0283) | 0.572 |
| suPAR | M0 | 0.014 (-0.0077 - 0.0356) | 0.206 | 0.0264 (0.0057 - 0.0471) | <0.001 | 0.0302 (0.0084 - 0.052) | 0.007 |
|  | M1 | 0.0065 (-0.0139 - 0.0269) | 0.535 | 0.0246 (0.0022 - 0.0471) | 0.012 | 0.0074 (-0.0133 - 0.0281) | 0.484 |
|  | M2 | 0.008 (-0.0128 - 0.0288) | 0.452 | 0.0041 (-0.0177 - 0.0259) | 0.031 | 0.0015 (-0.0215 - 0.0245) | 0.901 |
| PM_10_ nontraffic |  |  |  |  |  |  |  |
|  |  | Q2: β-Coefficient (95% CI) | P-value | Q3: β-Coefficient (95% CI) | P-value | Q4: β-Coefficient (95% CI) |  |
| Leukocyte count | M0 | -0.0033 (-0.0216 - 0.015) | 0.724 | -0.0017 (-0.0201 - 0.0166) | 0.854 | 0.0034 (-0.0149 - 0.0217) | 0.716 |
|  | M1 | -0.0093 (-0.0266 - 0.0081) | 0.295 | -0.0048 (-0.0223 - 0.0126) | 0.588 | -0.0059 (-0.0234 - 0.0115) | 0.506 |
|  | M2 | -0.0066 (-0.024 - 0.0109) | 0.461 | -0.0088 (-0.0267 - 0.0091) | 0.334 | -0.0099 (-0.028 - 0.0083) | 0.286 |
| NLR | M0 | 0.0011 (-0.0259 - 0.0281) | 0.935 | 0.0153 (-0.0118 - 0.0425) | 0.269 | 0.0023 (-0.0247 - 0.0294) | 0.866 |
|  | M1 | -0.0018 (-0.0294 - 0.0258) | 0.898 | 0.0088 (-0.0189 - 0.0365) | 0.533 | -0.004 (-0.0318 - 0.0237) | 0.776 |
|  | M2 | -0.0022 (-0.0305 - 0.0262) | 0.881 | 0.0069 (-0.0221 - 0.036) | 0.639 | -0.0081 (-0.0376 - 0.0213) | 0.588 |
| CRP | M0 | -0.023 (-0.1033 - 0.0572) | 0.574 | 0.0221 (-0.0588 - 0.1031) | 0.592 | -0.0374 (-0.1187 - 0.0438) | 0.366 |
|  | M1 | 0.0007 (-0.0757 - 0.0772) | 0.985 | 0.0376 (-0.0397 - 0.1149) | 0.341 | 0.001 (-0.0767 - 0.0787) | 0.979 |
|  | M2 | -0.0085 (-0.0861 - 0.0692) | 0.83 | 0.0192 (-0.0607 - 0.0992) | 0.637 | -0.0179 (-0.0995 - 0.0636) | 0.666 |
| Lp—PLA_2_ | M0 | 0.0283 (0.0061 - 0.0504) | 0.012 | 0.0172 (-0.0051 - 0.0395) | 0.13 | 0.0053 (-0.017 - 0.0276) | 0.64 |
|  | M1 | 0.0253 (0.003 - 0.0476) | 0.026 | 0.0137 (-0.0088 - 0.0362) | 0.231 | 0.0045 (-0.0181 - 0.027) | 0.699 |
|  | M2 | 0.0286 (0.0062 - 0.051) | 0.012 | 0.022 (-0.0011 - 0.045) | 0.062 | 0.0125 (-0.011 - 0.0359) | 0.297 |
| Ceruloplasmin | M0 | 0.02 (0.003 - 0.0369) | 0.021 | 0.0155 (-0.0016 - 0.0326) | 0.075 | 0.0175 (0.0004 - 0.0346) | 0.045 |
|  | M1 | 0.0189 (0.0018 - 0.0359) | 0.03 | 0.0156 (-0.0016 - 0.0329) | 0.076 | **0.0187 (0.0014 - 0.0361)** | **0.034** |
|  | M2 | 0.0144 (-0.003 - 0.0317) | 0.105 | 0.0121 (-0.0058 - 0.0299) | 0.186 | 0.0125 (-0.0058 - 0.0307) | 0.18 |
| Orosomucoid | M0 | 0.0288 (0.0063 - 0.0513) | 0.012 | 0.0389 (0.0163 - 0.0615) | 0.001 | 0.0149 (-0.0077 - 0.0375) | 0.197 |
|  | M1 | 0.0298 (0.0078 - 0.0517) | 0.008 | 0.0425 (0.0204 - 0.0646) | <0.001 | 0.0221 (-0.0001 - 0.0443) | 0.051 |
|  | M2 | 0.0318 (0.0098 - 0.0538) | 0.005 | 0.0398 (0.0172 - 0.0624) | 0.001 | 0.0212 (-0.0018 - 0.0441) | 0.07 |
| Haptoglobin | M0 | 0.0307 (-0.0062 - 0.0675) | 0.103 | 0.0282 (-0.009 - 0.0653) | 0.137 | 0.0157 (-0.0216 - 0.053) | 0.409 |
|  | M1 | 0.0289 (-0.0071 - 0.0649) | 0.115 | 0.0282 (-0.0083 - 0.0646) | 0.13 | 0.0121 (-0.0246 - 0.0488) | 0.517 |
|  | M2 | 0.0277 (-0.0089 - 0.0643) | 0.138 | 0.0227 (-0.015 - 0.0604) | 0.239 | 0.0056 (-0.0329 - 0.0441) | 0.775 |
| C3 | M0 | 0.0125 (-0.0043 - 0.0293) | 0.146 | 0.014 (-0.0029 - 0.0309) | 0.105 | 0.0022 (-0.0147 - 0.0191) | 0.796 |
|  | M1 | 0.0163 (0.0005 - 0.032) | 0.043 | 0.0172 (0.0013 - 0.0331) | 0.034 | 0.0154 (-0.0005 - 0.0313) | 0.057 |
|  | M2 | 0.0148 (-0.0009 - 0.0305) | 0.065 | 0.0158 (-0.0003 - 0.032) | 0.055 | 0.0094 (-0.007 - 0.0258) | 0.262 |
| alfa-1-antitrypsin | M0 | 0.0193 (0.0003 - 0.0382) | 0.046 | **0.0227 (0.0036 - 0.0418)** | **0.02** | 0.018 (-0.0011 - 0.037) | 0.065 |
|  | M1 | 0.0161 (-0.003 - 0.0351) | 0.098 | **0.0208 (0.0016 - 0.0401)** | **0.034** | 0.0161 (-0.0032 - 0.0354) | 0.102 |
|  | M2 | 0.0129 (-0.0067 - 0.0325) | 0.197 | 0.0154 (-0.0047 - 0.0355) | 0.134 | 0.0109 (-0.0096 - 0.0313) | 0.299 |
| suPAR | M0 | -0.0171 (-0.0388 - 0.0046) | 0.122 | 0.0041 (-0.015 - 0.0263) | 0.713 | -0.0148 (-0.0367 - 0.007) | 0.184 |
|  | M1 | -0.0189 (-0.0394 - 0.0015) | 0.069 | 0.0057 (-0.0173 - 0.0248) | 0.591 | -0.0135 (-0.0342 - 0.0072) | 0.202 |
|  | M2 | -0.0127 (-0.0331 - 0.0078) | 0.224 | 0.0038 ( -0.0134 – 0.0398 ) | 0.727 | -0.0145 (-0.0359 - 0.007) | 0.186 |
